# Distinct role of 5′UTR sequences in dendritic trafficking of BDNF mRNA: additional mechanisms for the BDNF splice variants spatial code

**DOI:** 10.1186/s13041-020-00680-8

**Published:** 2021-01-12

**Authors:** Andrea Colliva, Enrico Tongiorgi

**Affiliations:** Department of Life Sciences (Q Building), University of Trieste, Via Licio Giorgieri, 5, 34127 Trieste, Italy

**Keywords:** Neurotrophic factors, BDNF mRNA, RNA localization, Neuronal dendrites, Transport mechanism, Dendritic targeting elements, Fragile-X protein family, hnRNPs, Stress granules, P-bodies

## Abstract

The neurotrophin Brain-derived neurotrophic factor (BDNF) is encoded by multiple bipartite transcripts. Each BDNF transcript is composed by one out of 11 alternatively spliced exons containing the 5′untranslated region (UTR), and one common exon encompassing the coding sequence (CDS) and the 3′UTR with two variants (short and long). In neurons, BDNF mRNA variants have a distinct subcellular distribution, constituting a “spatial code”, with exon 1, 3, 5, 7 and 8 located in neuronal somata, exon 4 extending into proximal dendrites, and exon 2 and 6 reaching distal dendrites. We previously showed that the CDS encodes constitutive dendritic targeting signals (DTS) and that both the 3′UTR-short and the 3′UTR-long contain activity-dependent DTS. However, the role of individual 5′UTR exons in mRNA sorting remains unclear. Here, we tested the ability of each different BDNF 5′UTRs to affect the subcellular localization of the green fluorescent protein (GFP) reporter mRNA. We found that exon 2 splicing isoforms (2a, 2b, and 2c) induced a constitutive dendritic targeting of the GFP reporter mRNA towards distal dendritic segments. The other isoforms did not affect GFP-mRNA dendritic trafficking. Through a bioinformatic analysis, we identified five unique cis-elements in exon 2a, 2b, and 2c which might contribute to building a DTS. This study provides additional information on the mechanism regulating the cellular sorting of BDNF mRNA variants.

## Introduction

The neurotrophin Brain-derived neurotrophic factor (BDNF) is a key plasticity factor and its local translation at synapses is required for long term potentiation (LTP) [1]. BDNF is a highly conserved gene with a complex structure. In rodents, a common coding sequence (CDS) is spliced to one out of 11 upstream different 5′UTR alternative exons. Moreover, the 3′UTR contains two different polyadenylation sites which produce either a long or a short version of the 3′UTR, raising the number of resultant transcripts to a total of 22 variants [2]. Following a series of in vivo [3–5] and in vitro studies [3, 6–8] we and other groups have shown that in hippocampal pyramidal neurons, BDNF transcripts containing either exon 1, 3, 5, 7, or 8 are preferentially located in the soma, while transcripts with exon 4 extend into the proximal dendrites, and transcripts with exon 2 or 6 reach the distal dendritic compartment in an activity-dependent manner. Interestingly, in dentate gyrus (DG) hippocampal neurons in vivo, also the exon 3 transcript is targeted to the distal dendrites following *status epileticus* [5]. Based on these studies, we proposed that the differential subcellular distribution of BDNF transcripts endows neurons with a “spatial code” for a segregated expression of BDNF governed by the transcripts localized in the different subcellular compartments [3, 9]. Notably, variations in the local availability of the different BDNF transcripts in different cellular districts induce spatially restricted structural changes in the dendritic arbor [6, 10, 11].

The quest for the mechanisms regulating BDNF mRNA transport in dendrites has led to the discovery that the BDNF coding sequence (CDS) encodes a signal for constitutive dendritic targeting that requires the RNA-binding protein Translin [3]. However, the localization of BDNF mRNA in dendrites or its restriction to the soma depends on the combination of the CDS with the different 5′UTR sequences. The alternatively spliced 5′UTR exons act as selectivity signals that modify the constitutive dendritic targeting mediated by the coding sequence (CDS) of BDNF. Indeed, exon 1 and exon 4 cause the retaining of BDNF mRNA to the first 30% of the dendrite, while exons 2a, 2b, 2c and 6 allow its transport into the distal dendritic compartment [3, 6]. In addition, both short and long 3′ UTRs, display dendritic targeting in response to different stimuli: BDNF mRNAs with short 3′ UTR respond in vitro to depolarization and NT3, or to seizures in vivo, and require the RNA-binding proteins CPEB-1, -2 and ELAV-2, -4 [8, 12]. Instead, dendritic targeting of the long 3′UTR is triggered by neuronal activity or BDNF, and requires CPEB-1, ELAV-1, -3, -4, FXR2, and FMR1 [8, 12].

Despite these findings, one fundamental question remained unanswered: do the different 5′UTR splice variants contain targeting signals able to induce the sorting of BDNF mRNA to dendrites? The present study builds upon the above-cited works to evaluate how the different BDNF 5′UTR affect the subcellular localization of the green fluorescent protein (GFP) reporter mRNA, and identifies though a bioinformatic analysis the cis-acting elements present in the different BDNF 5′UTR variants.

## Results and discussion

**Fig. 1:**
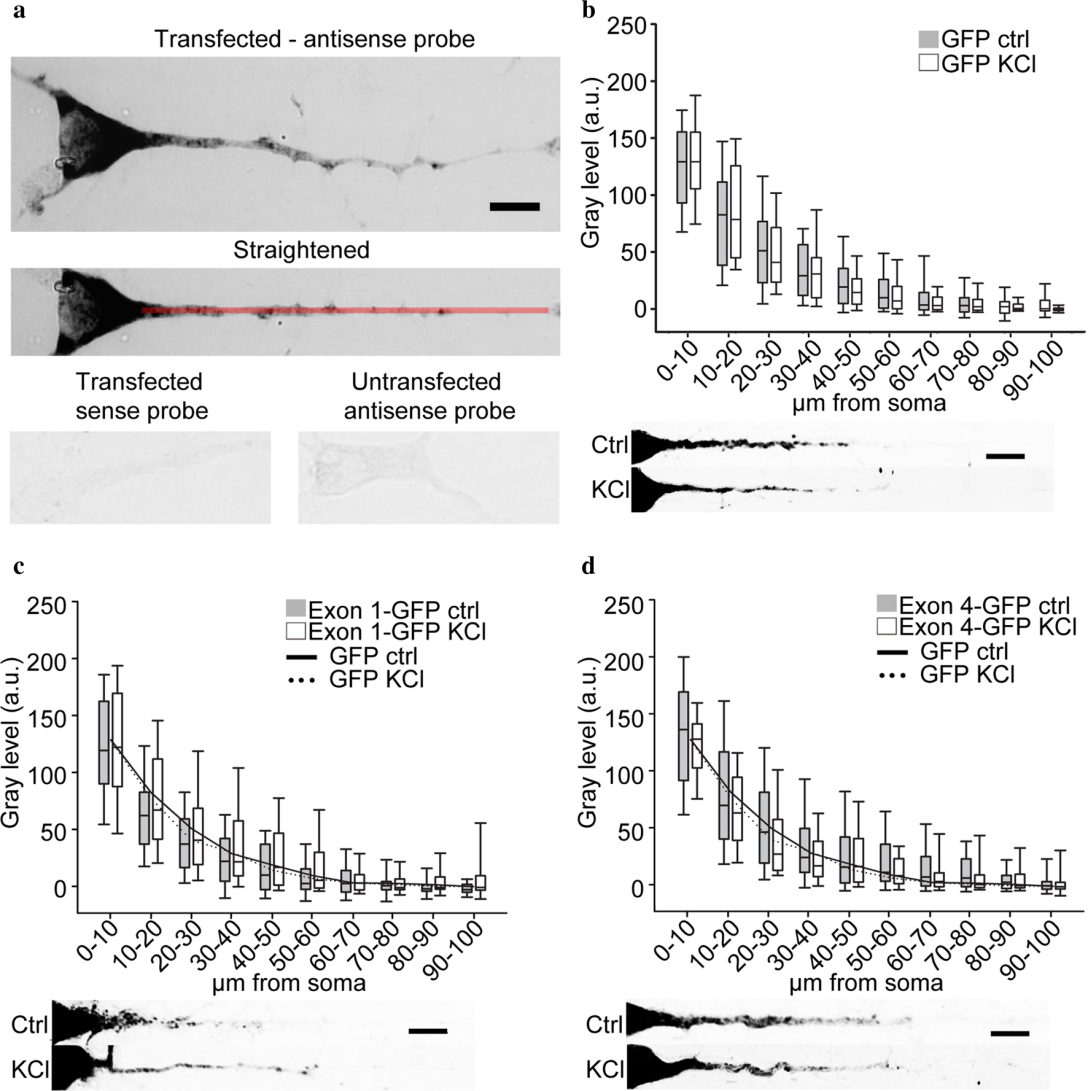
Trafficking of GFP, exon 1-GFP, and exon 4-GFP transcripts in hippocampal neurons. **a** Representative image of an in situ hybridization against the GFP coding sequence on one hippocampal pyramidal neuron transfected with pEGFP-N1 plasmid before (upper panel) and after straightening (central panel). Bottom panels represent two control in situ hybridization on pEGFP-N1 transfected neurons using GFP coding sequence sense probe (left) and on untransfected neurons using antisense probe. Scale bar: 10 μm. **b**–**d** Densitometric analysis on in situ hybridization of apical dendrites of neurons transfected with GFP (**b**), exon 1-GFP (**c**), and exon 4-GFP (**d**) untreated (gray boxes) or treated with 10 mM KCl for 3 h (white boxes). Below each graph, representative straightened neurons labeled by in situ hybridization are reported. Lines in graphs C and D represent the plot of the median gray value of GFP densitometric analysis untreated (solid line) and after KCl stimulation (dashed line). Scale bar: 10 μm

To investigate whether the eleven 5′UTR splicing isoforms of rat BDNF encode any constitutive or activity-dependent dendritic targeting signals (DTS), we cloned each 5′UTR region upstream the GFP gene, used as reporter transcript (for details see “Materials and methods”). Each chimaeric construct was transfected separately in cultured pyramidal hippocampal neurons at 6 days in vitro (DIV6) and then processed for in situ hybridization and densitometric analysis at DIV7. Figure 1a shows a representative in situ hybridization obtained using an antisense probe against GFP. The central panel of Fig. 1a shows the result of the application of the “Straighten” plug-in of ImageJ on the neuron shown at the top, leading to a linearized dendrite which was used for the densitometric analysis. The red line represents the region of interest in which the mean gray value was measured to evaluate the mRNA levels in the different compartments. The specificity of the in situ hybridization labeling was determined by using a sense probe for the GFP coding sequence on transfected neurons or a GFP antisense probe in non-transfected neurons. In both cases, the in situ signal obtained is indistinguishable from the background (Fig. 1a, bottom panel).

In agreement with our previous studies [3, 6], in situ labeling for the GFP mRNA which had no BDNF 5′UTR insert, was found only in the soma and proximal dendrites of transfected neurons, as confirmed by densitometric analysis (Fig. 1b, bottom panel; GFP ctrl). The GFP mRNA has largely been used as negative control in the literature on neuronal mRNA trafficking, because it does not contain active dendritic targeting elements. Gray bars represent the plot of the mean gray values of control (= untreated) neurons, binned in 10 μm compartments starting from soma up to the first 100 μm of the apical dendrite. In parallel cultures, GFP-transfected neurons were treated for 3 h with a 10 mM KCl solution to assess if GFP mRNA is targeted to dendrites in response to enhanced neuronal activity. Densitometric analysis on KCl-treated transfected neurons confirmed that GFP mRNA distribution was unaffected by KCl-induced depolarization (Fig. 1b, white bars; GFP KCl).

**Fig. 2:**
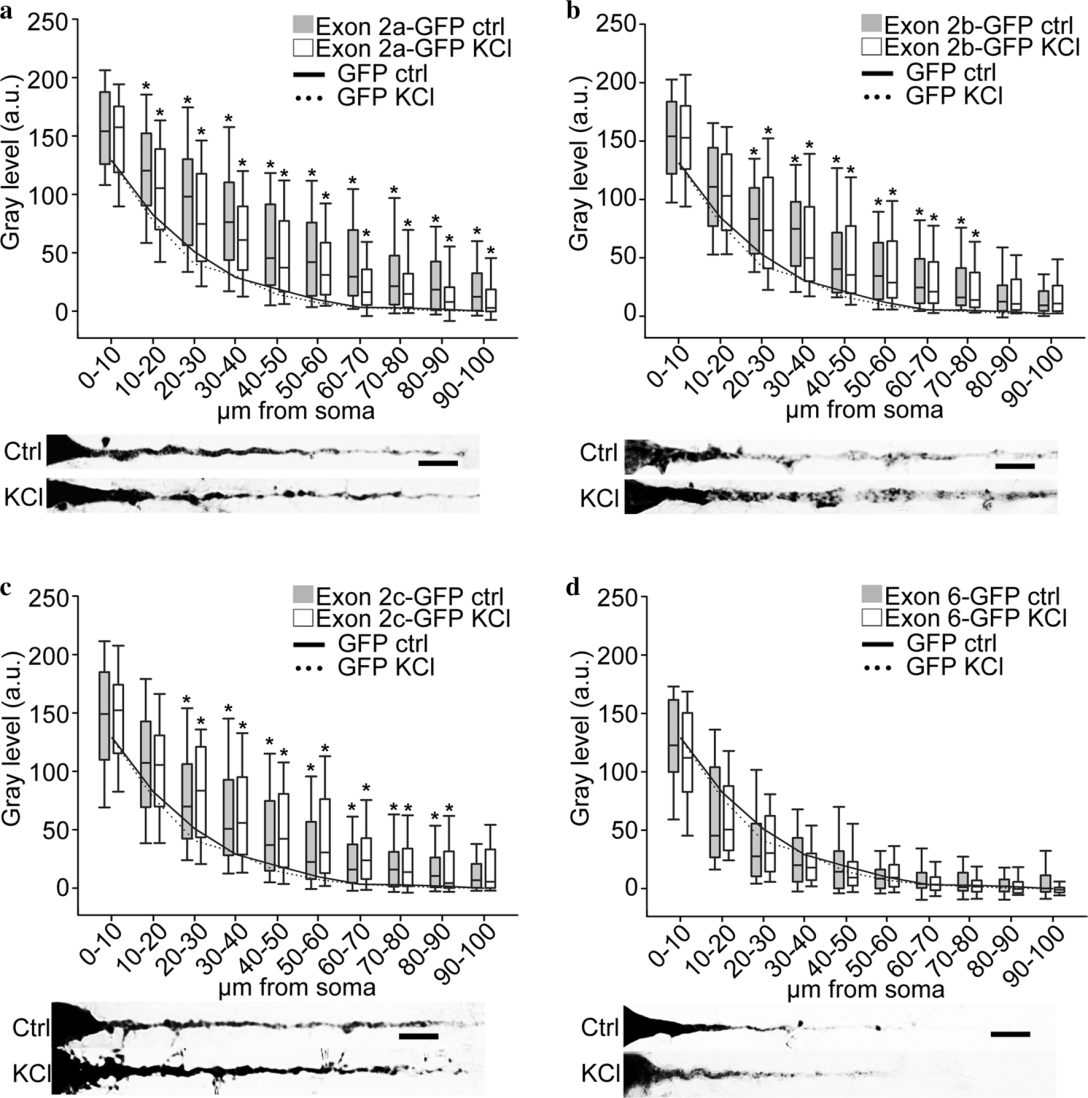
Trafficking of exon 2a-GFP, exon 2b-GFP, exon 2c-GFP, and exon 6-GFP transcripts in hippocampal pyramidal neurons. **a**–**d** Densitometric analysis on in situ hybridization of apical dendrites of pyramidal neurons transfected with exon 2a-GFP (**a**), exon 2b-GFP (**b**), exon 2c-GFP (**c**) and exon 6-GFP (**d**) untreated (gray boxes) or treated with 10 mM KCl for 3 h (white boxes). Below each graph, representative straightened neurons labeled by in situ hybridization are reported. Lines in graphs represent the plot of the median gray value of GFP densitometric analysis untreated (solid line) and after KCl stimulation (dashed line). Scale bars: 10 μm. *p ≤ 0.05 exon-GFP ctrl respect to GFP control and exon-GFP KCl vs GFP KCl

**Fig. 3:**
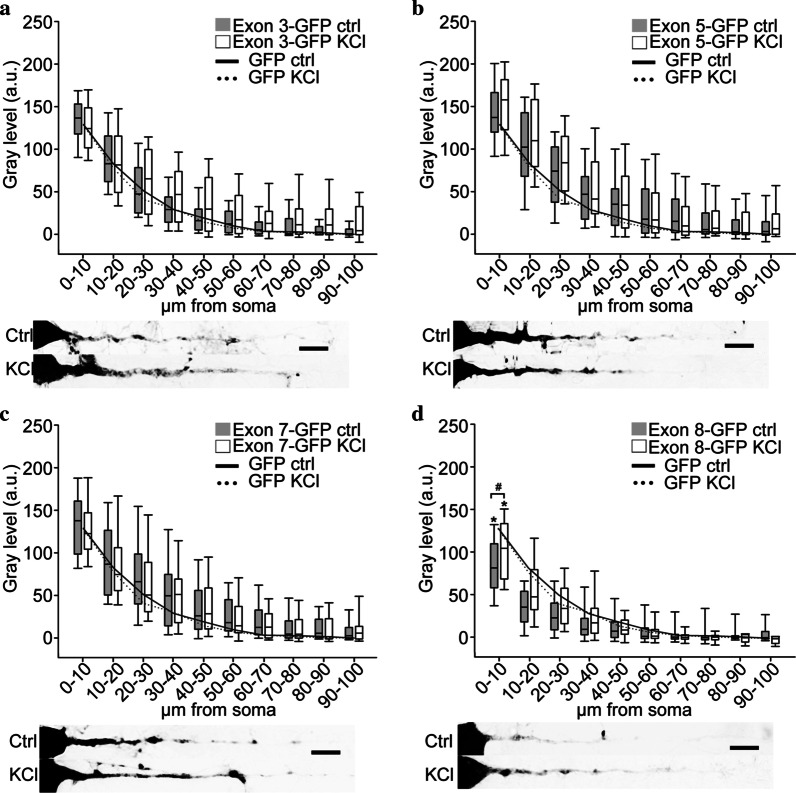
Trafficking of exon 3-GFP, exon 5-GFP, exon 7-GFP, and exon 8-GFP transcripts in hippocampal neurons. **a**–**d** Densitometric analysis of in situ hybridization on apical dendrites of neurons transfected with exon 3-GFP (**a**), exon 5-GFP (**b**), exon 7-GFP (**c**) and exon 8-GFP (**d**) untreated (gray boxes) or treated with 10 mM KCl for 3 h (white boxes). Below each graph, representative straightened neurons labeled by in situ hybridization are reported. Lines in graphs represent the plot of the median gray value of GFP densitometric analysis untreated (solid line) and after KCl stimulation (dashed line). Scale bars: 10 μm. *p ≤ 0.05 exon-GFP ctrl respect to GFP control and exon-GFP KCl vs GFP KCl. ^#^p ≤ 0.05 exon-GFP ctrl vs exon-GFP KCl

Using the same approach, we evaluated the distribution of chimaeric BDNF 5′ exons-GFP transcripts in untreated or depolarized neurons (raw data are provided in Additional file 1). As the in situ experiments for the control GFP plasmid shown in Fig. 1b (n = 4) were carried largely in parallel with those for the other chimaeric exon-GFP transcripts, the continuous and dashed lines shown in the graphs of experiments with exon-GFP chimeric constructs (Figs. 1, 2 and 3) are the plot of the median gray values of Fig. 1b. Exon 1 and exon 4, which were previously shown to inhibit the constitutive dendritic targeting of the BDNF CDS [3], did not alter the distribution of the GFP reporter mRNA, displaying a distribution similar to GFP mRNA alone (Fig. 1c, d). Exon 2 and 6 were previously demonstrated to be permissive for BDNF CDS-mediated dendritic targeting [3, 6]. Here, we found that only exon 2 variants induced a significant dendritic targeting of the GFP reporter transcript (Fig. 2a–c; *p < 0.05). These three exon 2 variants resulted to encode for a constitutively active DTS, because their dendritic localization was not affected by KCl-induced depolarization (Fig. 2a–c, white bars). Instead, exon 6 did not affect the GFP chimaeric transcript localization with respect to GFP alone, neither in unstimulated nor in depolarized conditions (Fig. 2d). We then evaluated the localization of the other BDNF 5′UTR splicing variants that were previously less characterized regarding their contribution to BDNF mRNA sorting. Exon 3-GFP chimaeric mRNA distribution in unstimulated condition was not significantly different with respect to GFP alone (Fig. 3a, gray bars) but the depolarization induced a weak, not statistically significant, transport into dendrites (Fig. 3a, white bars). Exon 5 and exon 7-GFP displayed a distribution similar to the GFP reporter transcript, in both conditions analyzed (Fig. 3b, c respectively). Exon 8-GFP resulted to be less abundant in dendrites of untreated neurons compared to the GFP transcript, especially in the first 40 μm (Fig. 3d, gray bars, exon 8-GFP CTRL; *p < 0.05). KCl depolarization significantly increased this transcript with respect to the untreated conditions in the soma, but not in dendrites (Fig. 3d, white bars, exon 8-GFP KCl; ^#^p < 0.05).

To get more insight into the possible mechanisms that make exon 2 different from the other BDNF 5′UTR exons, we first consider the sequence length, however we found no obvious specificity for exon 2 with respect to other sequences investigated in this study. In fact, the sequence length of the various exon 2 lay in between the others with exon 1 being the largest = 660 nt, exon 2a = 226 nt, exon 2b = 438 nt, exon 2c = 521 nt, exon 3 = 247 nt, exon 4 = 346 nt, exon 5 = 102 nt, exon 6 = 369 nt, exon 7 = 234 nt and exon 8 = 298 nt (Additional file 2). Thus, we favour the hypothesis that exon 2a, b, c may contain some specific recognition sites for RNA-Binding proteins (RBPs) involved in dendritic mRNA trafficking. To identify the RBP binding sites present in each BDNF 5′splicing isoform sequences, we performed a bioinformatic analysis on each BDNF 5′UTR variant (FASTA sequences in Additional file 2) using the RBPDB database (https://rbpdb.ccbr.utoronto.ca) containing 757 different RNA recognition motifs, and the RBPmap database (https://rbpmap.technion.ac.il) containing 114 human/mouse and 51 Drosophila melanogaster experimentally defined motifs. Each BDNF variant showed numerous putative RBP recognition sites (Table 1; Full details of recognition sequences and p values are reported in Additional files 3 and 4). Searching for motifs that could account for the constitutive DTS of exons 2a, 2b, and 2c, we focused on cis-elements common to all three variants but absent in the other BDNF 5′exons. We identified five sites unique to exon 2a, 2b and 2c, which are recognized by the RBPs Heterogeneous Nuclear Ribonucleoprotein A1 (HNRNPA1), Poly(A) Binding Protein Cytoplasmic 3 (PABPC3), Small Nuclear Ribonucleoprotein U1 Subunit 70 (SNRNP70), Zinc Finger CCCH-Type Containing 10 (ZC3H10), Zinc Finger CCHC-Type And RNA Binding Motif Containing 1 (ZCRB1) (highlighted in green in Table 1). Interestingly, all five recognition elements occur only once in each exon 2 sequence. Even if the algorithm indicated that the recognition motif for HNRNPA1 was present twice, given the large overlap of the two putative HNRNPA1-binding sites detected, we considered them as a single motif. Of note, according to the Protein Atlas (www.proteinatlas.org), all four RBPs are expressed in the brain, and HNRNPA1 and SNRNP70 show high expression levels in both cortex and hippocampus while PABPC3 and ZC3H10 show low expression levels in these brain areas.

**Table 1:**
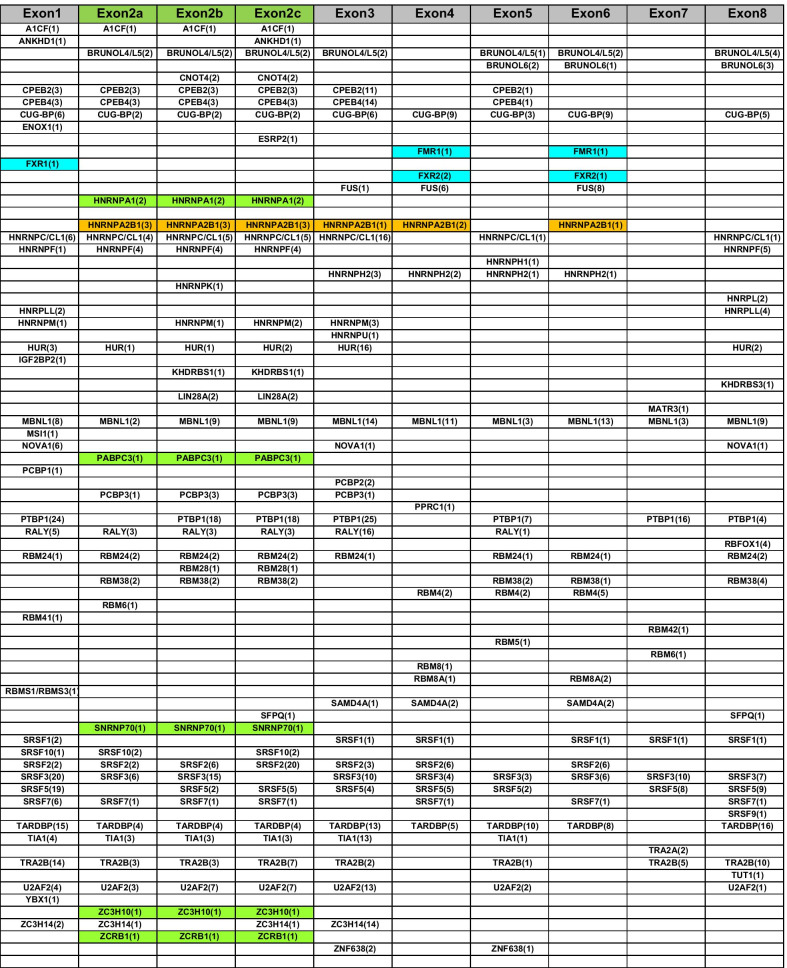
Identified RNA-binding protein cis-recognition motifs in rodents BDNF 5′UTRs

The RBPs unique to exons 2a, 2b, 2c are labeled in green. The HNRNPA2B1 is labeled in orange and the RBPs of the Fragile-X family are labeled in blue. Numbers in brackets indicate how many times the specified recognition sequence has been found in each BDNF 5'UTR variant

**Fig. 4:**
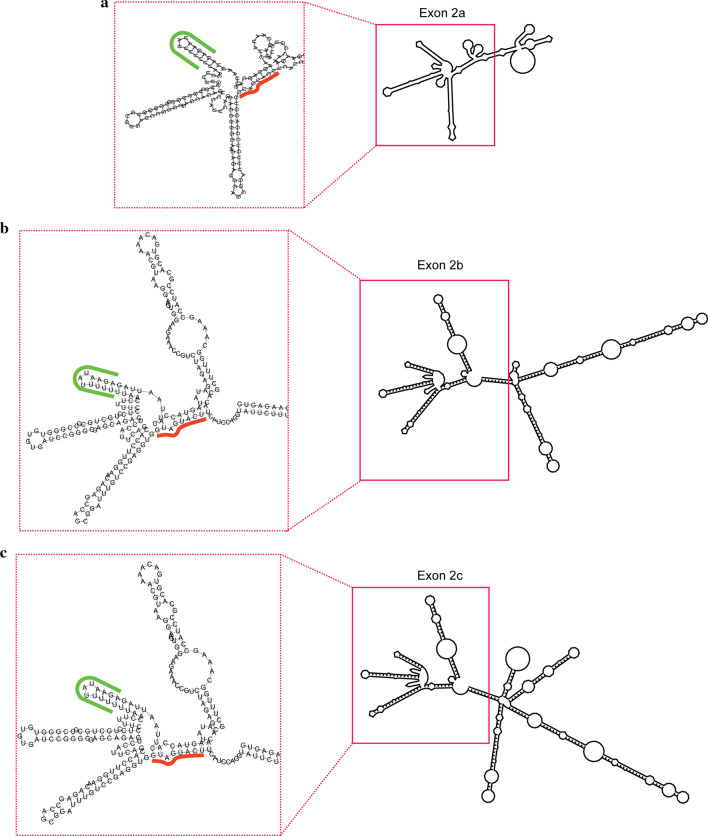
Predicted folding of exon 2 mRNA variants. **a**–**c** Predicted secondary structures using the RNAfold platform from the University of Vienna (https://rna.tbi.univie.ac.at/cgi-bin/RNAWebSuite/RNAfold.cgi) of *BDNF* mRNA 5′UTR splicing variants exon 2a (**a**), 2b (**b**) and 2c (**c**). The overall secondary structures of each exon are reported on the right. The insets on the left highlight a common region displaying a putative binding site for two clusters of RNA binding proteins: CPEB2/CPEB4, HNRNPC/CL1, HuR, TIA1 cluster (green curved line) and HNRNPA2/B1 cluster (orange straight line)

HNRNPA1 was previously identified as a component of neuronal mRNA-transporting granules by pull-down experiments with anti-Kinesin5 (KIF5) immunoprecipitating antibodies, together with several other RBPs involved in mRNA trafficking and translational regulation [13]. Therefore, it represents a strong candidate for being involved in the constitutive DTS for exons 2a, 2b, and 2c. Remarkably, in all exon 2 variants the recognition sequence for HNRNPA1 largely overlaps with the binding sequence for HNRNPA2/B1, another key factor in mRNA trafficking and translational regulation [14]. This recognition sequence is located in the central position of exon 2 RNA molecules and is quite distant from other regions that are characterized by long stem-loop structures containing recognition sites for other regulatory proteins (Fig. 4). HNRNPA2/B1 can form multimers when bound to the scaffolding protein tumor-overexpressed gene (TOG), which in turn, binds to multiple HNRNPA2 molecules [14]. Interestingly, we found one recognition site for HNRNPA2/B1 not only in all three exon 2 variants but also in exon 6, the other prominent BDNF mRNA with dendritic localization [3, 15] and exon 3, which we found to be enriched in the dendrites of hippocampal DG granule cells following *status epilepticus* [5] (Table 1, highlighted in orange). Since, another recognition site for HNRNPA2/B1 is also present in the CDS of BDNF (*data not shown*), a possible cooperative effect between multiple HNRNPA2/B1 molecules and HNRNPA1 is conceivable. It is worth noting that HNRNPA2/B1 was previously demonstrated to accumulate in the synapses of hippocampal neurons following stimulation with BDNF [16], so we cannot exclude that the HNRNPA2/B1 motif present in exon3 and 6 could specifically regulate the trafficking of these BDNF mRNA variants upon neurotrophic stimulation.

The other four unique RBP recognition sites present in exon 2 variants are likely involved in mRNA dendritic targeting and/or translational repression. PABPC3 is a less studied member of the poly(A)-binding protein family with a slightly lower affinity to poly(A) as compared to the best-characterized isoform PABP1 and belongs to the mRNA 3′End Processing Factors required for the process of polyadenylation involved in mRNA translation [17]. As PABP1 was shown to regulate the nuclear export of mRNAs towards the cytoplasm [18], and formation of P-bodies [19] it is plausible that also PABPC3 may play a role either in cytoplasmic mRNA trafficking or translational regulation, or both. SNRNP70 belongs to the family of small nuclear nucleoproteins that bind to the spliceosomal U1 RNA, forming the core of the spliceosome. SNRNP70 is essential for recognition of the pre-mRNA 5′splice-site and the subsequent spliceosome assembly. It has been shown that this protein is able to inhibit polyadenylation via direct interaction with the poly(A)-polymerase (PAP) [20]. Interestingly, recent studies have shown that SNRNP70 participates in the formation of detergent-insoluble aggregates in the brain of patients affected by Alzheimer's disease together with other well-characterized RNA-binding proteins such as TAR DNA-binding protein 43 (TDP-43) and fused in sarcoma (FUS), for which we found binding sites in exon 3 and exon 6, but not in exon 2 variants [21]. ZC3H10 is a CCCH zinc finger protein and belongs to a family of RNA-binding proteins that function as RNA metabolism regulators [22]. Its role in mRNA trafficking is unclear.

Another interesting information that we obtained from the bioinformatic analysis is related to the finding of recognition sequences for FXR1 in exon 1, and FXR2 and FMRP in exon 4 and 6. We have previously shown that these proteins provide signals for somatic retention of BDNF mRNA both in vitro and in vivo, and this retention signal is released by electrical activity, or BDNF application [12, 23]. Another observation pertains to the presence in exons 1, 2a, b, c, 3, and 5 of recognition sites for CPEB2 and CPEB4 which, in this context, do not appear generating any DTS. We have previously demonstrated that these recognition sites are also present in both short and long 3′ UTRs where they participate in the activity-dependent dendritic targeting of BDNF mRNA [8, 12]. The CPEB proteins are involved in multiple functions and their role is depending upon the reciprocal distance of their recognition sites and upon the distance of each site from the 3′ end [8]. Regarding the BDNF 5′UTRs, we favour the hypothesis that CPEB2-CPEB4 might be involved in the formation of P-bodies and stress granules together with another prominent stress granule protein, namely TIA1 [24], whose recognition site is also present in exons 1, 2a, b, c, 3, and 5. Stress granules (SGs) and processing bodies (P-bodies or PB) are membraneless ribonucleoprotein-based cellular compartments that are evolutionary conserved from fungi to vertebrates [25]. P-bodies, or processing bodies, are discrete cytoplasmic foci formed by phase separation within the cytoplasm of eukaryotic cells, consisting of many enzymes involved in mRNA turnover. They are composed of messenger ribonucleic acid-protein complexes containing a subset of enzymes and proteins involved in mRNA decay. In contrast, Stress Granules (SG) are dense aggregations in the cytoplasm composed of proteins and mRNAs that appear when the cell is under stress. They contain translationally stalled mRNA pre-initiation complexes and function as decision point for the mRNA fate. Accordingly, mRNAs present in SG can be sorted towards three different possible fates: long-term storage, degradation (in P-bodies) or re-initiation of translation [25]. Of note, the recognition sites for these RBPs are clustered within one distinctive stem-loop structure that is present in all exon 2 variants (Fig. 4). This stem-loop structure additionally harbours the recognition sites for HNRNPC/CL1 and HUR (also known as ELAV-like protein 1; see Fig. 4) that are also involved in the formation of P-bodies and stress granules [26] and are part of the 3′UTR signals involved in the dendritic trafficking of BDNF mRNA [12].

Our bioinformatic analysis of the BDNF 5′sequences identified only a few putative binding sites for RNA binding proteins (RBPs) involved in mRNA trafficking. This is in agreement with the current literature, reporting that most of the cis-elements found in the 5′UTRs are involved in mRNA alternative splicing, stability, and translation, while the regulatory sequences for mRNA trafficking are preferentially located in the CDS or in the 3′UTR [27, 28]. However, some important exceptions to this rule were reported. For instance, the 5′UTR of the BC1 untranslated RNA encodes a DTS organized in a stem-loop structure bound by the HNRNPA2 protein [29]. In conclusion, following a systematic analysis of the different BDNF 5′UTR variants, we can exclude an active role for exon 1, 3, 4, 5, 6, 7, and 8 in dendritic targeting of BDNF mRNA. In addition, we provide evidence that exon 2 variants encode a constitutively active dendritic targeting signal, possibly involving an HNRNPA1 and HNRNPA2/B1-mediated interaction between 5′UTRs and CDS of BDNF to regulate BDNF mRNA dendritic trafficking.

## Materials and methods

### Animal treatment

Animals were treated according to the institutional guidelines in compliance with national laws (D.lgs. 26/2014, art.1/04), the European Council Directive 86/609, and NIH Guide for the care and use of laboratory animals. The present study has been carried out under project authorization N. NO88TON18 from the Italian Ministry of Health. Animals were housed in groups under standard conditions (12:12 h light/dark cycle, ambient temperature 23°C, access to food and water ad libitum).

### Primary hippocampal neuronal cultures

Primary rat hippocampal neuronal cultures were prepared from P0–P1 Wistar rat pups as previously described [30]. In brief, hippocampi were dissected and collected into tubes with ice-cold Hanks balanced salt solution (HBSS; 4.2 mM NaHCO_3_, 12 mM Hepes, 33 mM D-glucose, 2 mM Kinurenic acid and 0.95% Hank's salt powder, all purchased from Sigma). Tissue was digested for 8 min with trypsin 0.25% (Euroclone) in HBSS solution, then an equal volume of Dulbecco's Modified Eagle Medium (DMEM; Euroclone) supplemented with penicillin/streptomycin (Euroclone) and 10% fetal bovine serum (FBS, Euroclone) was added to stop enzymatic digestion. Digested tissue was centrifuged at room temperature for 5 min at 800 rpm, the supernatant was removed and replaced with pre-warmed DMEM + 10% FBS. Tissue was homogenized by pipetting, the homogenate was filtered through a 0.40 μm cell strainer (Falcon DB). Cells were plated at a density of 1 × 10^5^ cells in a 24 multi-well plate (Sarstedt) on 12 mm cover glasses (Sacco) pre-treated with 0.1% poly-ornithine (Sigma) and coated with 2% Matrigel (BD science). After 1 h, the culture medium was replaced with fresh pre-warmed Neurobasal (Invitrogen) supplemented with B-27 (Invitrogen) and penicillin–streptomycin. Medium supplemented with 0.5 μM Cytosine β-d-arabinofuranoside (Sigma) was replaced at days in vitro (DIV) 2 to stop non-neuronal cell proliferation. Half of the medium was changed twice a week.

### Cloning and generation of BDNF 5′UTRs exons-pEGFP-N1 constructs

The different 5′UTR isoforms (exon 1, 2a, 2b, 2c, 3, 4, 5, 6, 7, 8—see Additional file 1) of rat BDNF gene were cloned into a pEGFP-N1 vector (Clonetech) to obtain the expression of the chimaeric transcript 5′UTR BDNF exon-GFP coding sequence. Total RNA was extracted from adult rat brain using TRIzol (Thermofisher) and retro-transcribed to cDNA using Superscript-II transcriptase (Promega). Forward primers for 5′UTR exons cloning introduced a XhoI restriction site into the amplicon, while reverse primers introduced an AgeI restriction site. Amplification of the specific exon sequences were obtained using Phusion high-fidelity DNA polymerase (Finnzymes). Primers and PCR conditions are reported in Table 2. Amplified sequences were isolated on agarose gel using GenElute Gel extraction kit (Sigma) and cloned into pEGFP-N1 vector digested with XhoI and AgeI restriction enzymes (New England Biolabs) and ligase reaction was performed using T4-DNA ligase (New England Biolabs).

**Table 2:** Primers and PCR conditions for BDNF 5′UTR cloning

	Primer forward	Primer reverse	PCR conditions			
Exon I	AATTCTCGAGTAAAGCGGTAGCCGGCTGGTGCAGG	GTCTACCGGITTTGCTGTCCTGGAGACTCAGTGTC	Denaturation	98 °C	10 s	31 cycles
			Annealing	57 °C	20 s	
			Extension	72 °C	40 s	
Exon IIa	GATCCTCGAGGCTTTGGGAAAGCCATCCGCACGTGAC	GCCACCGGTCTGGATGAAGTACTACCACCTCGGAC	Denaturation	98 °C	10 s	31 cycles
			Annealing	58 °C	20 s	
			Extension	72 °C	35 s	
Exon llb	GATCCTCGAGGCTTTGGCAAAGCCATCCGCACGTGAC	GTTACCGOTOCAGCTTGCCAAGAGTCTATTCCAG	Denaturation	98 °C	10 s	31 cycles
			Annealing	58 °C	20 s	
			Extension	72 °C	35 s	
Exon llc	GATCCTCGAGGCTTTGGCAAAGCCATCCGCACGTGAC	GA/\ACCGGTCTTCTTTGCGGCTTACACCACCCCGGTGGCTAG	Denaturation	98 °C	10 s	31 cycles
			Annealing	58 °C	20 s	
			Extension	72 °C	35 s	
Exon III	AGGCTCGAGGCCCTCACGATTCTCGCTGGATAG	GCTAACCGGTCTGGGCTCAATGAAGCATCCAGCCC	Denaturation	98 °C	15 s	31 cycles
			Annealing	55 °C	15 s	
			Extension	68 °C	20 s	
Exon IV	AAITCTCGAGACCCACTTTCCCATTCACCGAGG	GACTACCGGTCAGTCACTACTTGTCAAAGTAAACATCAAGGC	Denaturation	98 °C	10 s	31 cycles
			Annealing	58 °C	20 s	
			Extension	72 °C	20 s	
Exon V	GACTCTCGAGAAACCATAACCCCGCACACTCTGTGTAGTTTCATTGTGTGTTCG	GACTACCGGTCTTCCCGCACCTTCCCGCACCACAGAGCTAGAAAAAGCGAACACAC	Denaturation	98 °C	10 s	31 cycles
			Annealing	58 °C	20 s	
			Extension	72 °C	15 s	
Exon VI	GACTCTCGAGCCAATCGAAGCTCAACCGAAGAGC	GATCACCGGTCTCAGGGTCCACACAAAGCTCTCGG	Denaturation	98 °C	10 s	31 cycles
			Annealing	57.5 °C	20 s	
			Extension	72 °C	15 s	
Exon VII	GACTCTCGAGCACTGTCACCTGCTTTCTAGGGAGTATTACC	GTACACCGGTCTCCCGGATGAAAGTCAAAACTTTCACTTCCTCTGGAGG	Denaturation	98 °C	10 s	31 cycles
			Annealing	58 °C	20 s	
			Extension	72 °C	20 s	
Exon VIII	GTCACTCGAGGTTATAGAGTTGGATGCAAGCGTAACCCG	GCATACCGGTGACACCATTTCAGCAATCGTTTGTTCAGCTCC	Denaturation	98 °C	10 s	31 cycles
			Annealing	58 °C	20 s	
			Extension	72 °C	35 s	

### Constructs transfection

Rat hippocampal cultured neurons were transfected at DIV6 with 2 μl of Lipofectamine 2000 (Invitrogen) and 1 μg of chimaeric plasmid DNA (exon 1, 2a, 2b, 2c, 3, 4, 5, 6, 7, or 8-GFP constructs) as previously described [6] with slight modifications. Briefly, after 1 h, medium with transfectant reagent was removed and replaced with fresh medium. After 24 h from transfection, cells were depolarized for 3 h with a isosmotic solution containing 10 mM KCl, 1.8 mM CaCl_2_, 1.6 mM MgSO_4_, 100 mM NaCl, 26 mM NaHCO_3_, 1 mM NaH_2_PO_4_, 30 mM HEPES and 0.7% D-glucose (all reagents were from Sigma). At the end of the depolarization stimulus, treated and untreated cells were fixed in a 4% paraformaldehyde (PFA) in PBS at room temperature for 15 min and immediately assayed for in situ hybridization.

### In situ hybridization

In situ hybridization was performed as previously described [31]. Cells were fixed for 15 min at RT in 4% PFA in PBS, washed in PBS 0.1% Tween20 (Sigma) (PBST), and permeabilized in absolute ethanol for 15 min at − 20°C. After rehydration with increasing concentration of PBST (50% ethanol/50% PBST, 30% ethanol/70% PBST, and finally PBST), cells were hybridized with approximately 50–100 ng/coverslip of antisense or sense probes for GFP or BDNF coding sequence. Before probe hybridization, coverslips were equilibrated at 55°C in hybridization buffer containing 20 mM Tris–HCl pH 7.5 (Sigma), 300 mM NaCl (Sigma), 1 mM EDTA (Sigma), 0.5 mg/ml polyadenylic acid (Sigma), 0.5 mg/ml salmon sperm (Labtek Eurobio), 1× Denhardt's solution, 100 mM Dithiothreitol (DTT, Sigma) and 50% deionised formamide (Sigma). After 1 h, the hybridization buffer was removed and replaced with the hybridization mix (hybridization buffer + 10% dextran sulfate, Sigma) containing probes. Coverslips were incubated overnight at 55°C. Then, hybridization mix was removed, coverslips were washed with 2× Sodium Saline Citrate (Sigma) buffer (150 mM NaCl, 15 mM C_6_H_5_Na_3_O_7_) containing 0.1% Tween20 (SSCT 2×) and 50% formamide at 55°C twice, then with SSCT 2× at 55°C once and finally in SSCT 0.1× at 60°C twice. Coverslips were incubated with PBST 5% fetal bovine serum (FBS; Euroclone) for 1 h at room temperature to block unspecific binding of primary antibodies, then incubated with anti-digoxigenin alkaline phosphatase-conjugated antibody (Roche) in PBST 5% FBS for 2 h at room temperature. After washing with PBST to remove the excess of unbound antibody, hybridized probes were detected by developing in situ signal with 70 mg/ml 4-nitroblue tetrazolium (NBT, Labtek Eurobio) and 50 mg/ml 5-bromo-4-chloro-3-indolyl-phosphate (BCIP, Sigma) in a buffer solution containing 100 mM Tris–HCL pH 9.5, 50 mM MgCl_2_, 100 mM NaCl and 1 mM Levamisole (Sigma). Developing was carried out in the dark and time of incubation was determined empirically. The reaction was stopped by removing developing buffer and replacing it with a stop solution containing 10 mM Tris–HCl pH 8.0 and 1 mM EDTA pH 8.0. Coverslips were washed with PBS and deionized water and then mounted with Mowiol (Sigma).

### Digoxigenin-labeled probes synthesis

Probe synthesis was performed as previously described [3]. The open reading frame of GFP was subcloned into pBluescript and digoxygenin (DIG)-labelled probes were synthesized with DIG-RNA labeling kit (Roche Diagnostics) using linearized plasmids as templates, according to the manufacturer’s instructions. GFP sense and antisense probe were fragmented through alkaline hydrolysis in a carbonate buffer (40 mM NaHCO_3_, 60 mM Na_2_CO_3_, pH 10.2) to increase probe penetration into cells.

### Densitometric analysis on non-radioactive in situ hybridization

Non-radioactive in situ hybridization for GFP coding sequence was analyzed by viewing stained cultures under bright-field illumination as previously described [3] using a Nikon AXM1200 digital camera on a Nikon E800 Microscope with interference contrast-equipped lens (60× magnification) and then analyzed with the image analysis program ImageJ (NIH). Apical dendrites of *bona fide* pyramidal neurons identified by morphological criteria have been linearized using the “Straighten” plugin [32] as previously described [33]. Dendrites were traced conservatively, starting from the base of dendrite after soma and up to the point in which in situ labeling was distinguishable. The background level for each image was determined by evaluation of the mean gray value in the distal portion of apical dendrites of transfected neurons in which the in situ labeling was absent. At least 50 neurons from 3 or 4 independent cultures were analyzed for GFP, exon 1, 2a, 2b, 2c, 4, and 6 densitometric analysis, while at least 30 neurons from 2 to 4 independent cultures were analyzed for exon 3, 5, 7 and 8.

### Bioinformatic analysis

The research of putative cis-elements for RNA Binding Proteins on rat BDNF 5′UTR sequence was performed using RBPDB (https://rbpdb.ccbr.utoronto.ca and RBPmap (https://rbpmap.technion.ac.il) databases). A threshold of 0.8 was set for RBPDB database, while high stringency level and conservation filter were set for RBPPmap, then FASTA sequences were scanned by the two algorithms separetely. The 5′UTR two-dimensional conformation was investigated with the platform RNAfold from the University of Vienna (https://rna.tbi.univie.ac.at/cgi-bin/RNAWebSuite/RNAfold.cgi) [34, 35].

### Statistical analysis

Statistical data analysis and graph generation were carried out using Sigma Plot 11 software (Systat Software Inc.). The 5′UTR-GFP densitometries were analyzed with Krustal-Wallis ANOVA on ranks for exon-GFP untreated or depolarized versus GFP control or depolarized condition in all exon-GFP experiments.

## Supplementary information

**Additional file 1. **Row data of the densitometric analysis of the in situ hybridization for the different 5′UTR BDNF exons.

**Additional file 2.** FASTA sequences of the 5′UTR BDNF exons used in the bioinformatic analysis.

**Additional file 3.** Recognition sequences and p values of the bioinformatic analysis of each 5’UTR BDNF exon using RBPDB database.

**Additional file 4.** Recognition sequences and p values of the bioinformatic analysis of each 5’UTR BDNF exon using RBPmap database.

## Data Availability

All data generated or analyzed during this study are included in this published article and its additional information files.

## Abbreviations

A1CF: APOBEC1 complementation factor
ANKHD1: Ankyrin repeat and KH domain-containing protein 1
BCIP: 5-Bromo-4-chloro-3-indolyl-phosphate
BDNF: Brain-derived neurotrophic factor
BRUNOL4: Bruno-like 4
BRUNOL5: Bruno-like 5
BRUNOL6: Bruno-like 6
CDS: Coding sequence
CNOT4: CCR4-NOT transcription complex, subunit 4
CPEB2: Cytoplasmic polyadenylation element-binding protein 2
CPEB4: Cytoplasmic polyadenylation element-binding protein 4
CTRL: Control
CUG-BP: CUG triplet repeat, RNA binding protein
DIG: Digoxygenin
DMEM: Dulbecco's Modified Eagle Medium
EDTA: Ethylene-diamino tetra-acetate
ENOX1: Ecto-NOX Disulfide-Thiol Exchanger 1
ESRP2: Epithelial Splicing Regulatory Protein 2
FBS: Fetal bovine serum
FMR1: Fragile X mental retardation 1
FXR1: FMR1 Autosomal Homolog 1
GFP: Green fluorescent protein
HBSS: Hanks balanced salt solution
HNRNPA1: Heterogeneous Nuclear Ribonucleoprotein A1
HNRNPA2/B1: Heterogeneous Nuclear Ribonucleoprotein A2/B1
HNRNPA2: Heterogeneous Nuclear Ribonucleoprotein A2
HNRNPC/CL1: Heterogeneous nuclear ribonucleoprotein C-like 1
HNRNPF: Heterogeneous Nuclear Ribonucleoprotein F
HNRNPH1: Heterogeneous Nuclear Ribonucleoprotein H1
HNRNPH2: Heterogeneous Nuclear Ribonucleoprotein H2
HNRNPK: Heterogeneous Nuclear Ribonucleoprotein K
HNRNPM: Heterogeneous Nuclear Ribonucleoprotein M
HNRNPU: Heterogeneous Nuclear Ribonucleoprotein U
HNRPL: Heterogeneous Nuclear Ribonucleoprotein L
HNRPLL: Heterogeneous Nuclear Ribonucleoprotein L-Like
HuR: Human antigen R
IGF2BP2: Insulin-Like Growth Factor 2 MRNA Binding Protein 2
KHDRBS1: KH RNA Binding Domain Containing, Signal Transduction Associated 1
KHDRBS3: KH RNA Binding Domain Containing, Signal Transduction Associated 3
LIN28A: Lin-28 Homolog A
LTP: Long term potentiation
MATR3: Matrin 3
MBNL1: Muscleblind-Like Splicing Regulator 1
mRNA: Messenger ribonucleic acid
MSI1: Musashi RNA Binding Protein 1
NBT: 4-Nitroblue tetrazolium
NOVA1: RNA-binding protein Nova-1
PABPC3: Poly(A) Binding Protein Cytoplasmic 3
PAP: Poly(A)-polymerase
P-bodies: Processing bodies
PBS: Phosphate-buffer saline
PBST: Phosphate-buffer saline + Tween20
PCBP1: Poly(RC) Binding Protein 1
PCBP2: Poly(RC) Binding Protein 2
PCBP3: Poly(RC) Binding Protein 3
PCR: Polymerase chain reaction
PTBP1: Polypyrimidine tract-binding protein 1
RALY: RNA-binding protein Raly
RBFOX1: RNA Binding Fox-1 Homolog 1
RBM24: RNA Binding Motif Protein 24
RBM28: RNA Binding Motif Protein 28
RBM38: RNA Binding Motif Protein 38
RBM4: RNA Binding Motif Protein 4
RBM5: RNA Binding Motif Protein 5
RBM6: RNA Binding Motif Protein 6
RBM8A: RNA Binding Motif Protein 8A
RBM41: RNA Binding Motif Protein 41
RBM42: RNA Binding Motif Protein 42
RBMS1/RBMS3: RNA Binding Motif Single Stranded Interacting Protein 1/RNA Binding Motif Single Stranded Interacting Protein 3
RBP: RNA-binding protein
RT: Room temperature
SAMD4A: Sterile Alpha Motif Domain Containing 4A
SFPQ: Splicing Factor Proline and Glutamine Rich
SNRNP70: Small Nuclear Ribonucleoprotein U1 Subunit 70
SRSF1: Serine and Arginine Rich Splicing Factor 1
SRSF2: Serine and Arginine Rich Splicing Factor 2
SRSF3: Serine and Arginine Rich Splicing Factor 3
SRSF5: Serine and Arginine Rich Splicing Factor 5
SRSF7: Serine and Arginine Rich Splicing Factor 7
SRSF9: Serine and Arginine Rich Splicing Factor 9
SRSF10: Serine and Arginine Rich Splicing Factor 10
SSC: Sodium Saline Citrate
SSCT: Sodium Saline Citrate + Tween20
TARDBP: TAR DNA-binding protein
TIA1: Tia1 cytotoxic granule-associated RNA binding protein
TOG: Tumor overexpressed gene;
TRA2A: Transformer 2 Alpha Homolog
TRA2B: Transformer 2 Beta Homolog
TUT1: Terminal Uridylyl Transferase 1, U6 SnRNA-Specific
U2AF2: U2 Small Nuclear RNA Auxiliary Factor 2
UTR: Untranslated region
YBX1: Y-Box Binding Protein 1
ZC3H10: Zinc Finger CCCH-Type Containing 10
ZC3H14: Zinc Finger CCCH-Type Containing 14
ZCRB1: Zinc Finger CCHC-Type and RNA Binding Motif Containing 1
ZNF638: Zinc Finger Protein 638
