# Supplementary material for: Distinct role of 5′UTR sequences in dendritic trafficking of BDNF mRNA: additional mechanisms for the BDNF splice variants spatial code
Source: Mol Brain. 2021 Jan 12;14:10. doi: 10.1186/s13041-020-00680-8 (PMC7805101; doi:10.1186/s13041-020-00680-8)
Supplement: Supplementary file 2 — Additional file 2. FASTA sequences of the 5′UTR BDNF exons used in the bioinformatic analysis. [file 13041_2020_680_MOESM2_ESM.docx]

>EXON1

TAAAGCGGTAGCCGGCTGGTGCAGGAAAGCAACAAGTTCCCCAGCGGTCTTCCCGCCCTAGCCTGACAAGGCGAAGGTTTTCTTACCTGGCGACAGGGAAATCTCCTGAGCCGAGCTCATCTTTGCCACAGCCCCAGGTGTGACCTGAGCAGTGGGCAAAGGAGCGGCGTGCAAATTGGATTATTTGTATGGGGGTACTCTGAAACTCCCTCACTTTTTCTGGGAACTTTTTGTGCTAGGGCGCAGTGACAGGCGTTGAGAAAGCTGCTTCAGGAAACGCCCGCTATATAGCAGGGCAGTTGGACAGTCATTGGTAACCTCGCTCATTCATTAGAATCACGTAAGAACTCAAAGGGAAACGTGTCTCTCAGAATGAGGGCGTTTGCGTAAATCTATAGGTTTTTCAACATCGATGCCAGTTGCTTTGTCTTCTGTAATCGCCAAGGTGGATGAGAGTTGAAGCTTGCGGATATTGCAAAGGGTTATTAGATTCATAAGTCACACCAAGTGGTGGGCGATCCACTGAGCAAAGCCGAACTTCTCACATGATGACTTCAAACAAGACACATTACCTTCCAGCATCTGTTGGGGAGACGAGATTTTAAGACACTGAGTCTCCAGGACAGCAAAGCCACAATGTTCCACCAGGTGAGAAGAGTG

>EXON2A

GCTTTGGCAAAGCCATCCGCACGTGACAAAACGTAAGGAAGTGGAAGAAACCGTCTAGAGCAATATCAAGTACCACTTAATTAGAGAATATTTTTTTAACCTTTTCCTCCTGCTGCGCCGGGTGTGTGATCCGGGCGAGCAGAGTCCATTCAGCACCTTGGAACAGAGCCAGCGGATTTGTCCGAGGTGGTAGTACTTCATCCAGTTCCACCAGGTGAGAAGAGTG

>EXON2B

GCTTTGGCAAAGCCATCCGCACGTGACAAAACGTAAGGAAGTGGAAGAAACCGTCTAGAGCAATATCAAGTACCACTTAATTAGAGAATATTTTTTTAACCTTTTCCTCCTGCTGCGCCGGGTGTGTGATCCGGGCGAGCAGAGTCCATTCAGCACCTTGGAACAGAGCCAGCGGATTTGTCCGAGGTGGTAGTACTTCATCCAGGTATTCTTTTCCTCGCTGTCAAGCCAACCCGGTGTCGCCCTTAAAAAGCGTCTTTTCCGAGGTTCGGCTCACACTGAGATCGGGGCTGGAGAGAGAGTCAGATTTTGGAGCGGAGCGTTTGGAGAGCCAGCCCCAGTTTGGTCCCCTCATTGAGCTCGCTGAAGTTGGCTTCCTAGCGGTGTAGGCTGGAATAGACTCTTGGCAAGCTCCGGTTCCACCAGGTGAGAAGAGTG

>EXON2C

GCTTTGGCAAAGCCATCCGCACGTGACAAAACGTAAGGAAGTGGAAGAAACCGTCTAGAGCAATATCAAGTACCACTTAATTAGAGAATATTTTTTTAACCTTTTCCTCCTGCTGCGCCGGGTGTGTGATCCGGGCGAGCAGAGTCCATTCAGCACCTTGGAACAGAGCCAGCGGATTTGTCCGAGGTGGTAGTACTTCATCCAGGTATTCTTTTCCTCGCTGTCAAGCCAACCCGGTGTCGCCCTTAAAAAGCGTCTTTTCCGAGGTTCGGCTCACACTGAGATCGGGGCTGGAGAGAGAGTCAGATTTTGGAGCGGAGCGTTTGGAGAGCCAGCCCCAGTTTGGTCCCCTCATTGAGCTCGCTGAAGTTGGCTTCCTAGCGGTGTAGGCTGGAATAGACTCTTGGCAAGCTCCGGGTTGGTATACTGGGTTAACTTTGGGAAATGCAAGTGTTTATCTCCAGGATCTAGCCACCGGGGTGGTGTAAGCCGCAAAGAAGTTCCACCAGGTGAGAAGAGTG

>EXON3

ACCTCTCCATCGCCCTCACGATTCTCGCTGGATAGTTCTTTATGTTTTGGGTGATTTTTGTTTTTTGTTTGTTTGGTTTTTTTTTTTTTTAATTTTTACCCCTTTCTATTTTCCCTCCCCGAGAGTTCCGGGCTCTGGCTTGGAGGGCTCCTGCTTTCTCAAGGGAAGGGGAGCCGCTGAGACTGCGCTCCACTCCCTGCCGGGCTGGATGCTTCATTGAGCCCAGTTCCACCAGGTGAGAAGAGTG

>EXON4

ACCCACTTTCCCATTCACCGAGGAGAGGACTGCTCGCGCTGCCGCTCCCCCCACCCACCCCCGGCGAGCTAGCATGAAATCTCCCAGTCTCTGCCTAGATCAAATGGAGCTTCTCACTGAAGGCGTGCGAGTATTACCTCCGCCATGCAATTTCCACTATCAATAATTTAACTTCTTTGCTGAAGAACAGGAGTACATATCGGCCACCAAAGACTCGCCCCCCTCCCCCTTTTAACTGAAGAGAAGGGGAAATATATAGTAAGAGTCTAGAACCTTGGGGACCGGTCTTCCCCAGAGCAGCTGCCTTGATGTTTACTTTG ACAAGTAGTGACTGAAAAAG

>EXON5

AAACCATAACCCCGCACACTCTGTGTAGTTTCATTGTGTGTTCGCTTTTTCTAGCTCTGTGGTGCGGGAAGGTGCGGGAAGTTCCACCAGGTGAGAAGAGTG

>EXON6

CCAATCGAAGCTCAACCGAAGAGCTAAATAATGTCTGACCCCAGTGCCTGGCGCTGGCTGAGCTCTGGGTGCCCGCCGCTGCCGCCGCGCCGGGGCGCACCCGCTGGCTGGCTGTCGCACGGTCCCCATTGCGCCCGGGACTCCCCGGCTTGGAGAAGGAAACCGCCTGGGGCGGCGCGCCACCTCCGCCTGGCAGGCTTTGATGAGACCGGGTTCCCTCAGCTCGCCACCGCTGCTTTGGGGCAGACGAGAAAGCGCACGGGGCCCAGGGCAGGGCGCAGGGACCAGGAGCGTGACAACAATGTGACTCCACTGCCGGGGATCCGAGAGCTTTGTGTGGACCCTGAGTTCCACCAGGTGAGAAGAGTG

>EXON7

CACTGTCACCTGCTTTCTAGGGAGTATTACCAAAGCTTCTTACAAGTCCAAGGTCAACATAACTAGGAACTTGGGATCATTCTTGTCACTGGGACCTGAAAGGGTCTGCGGAACTCCAGGTCCCACAGCTTGTATCCGACCCTCTCTGTCCATCCAGCGCACCTCTTTAGGCATCCTCCAGAGGAAGTGAAAGTTTTGACTTTCATCCGGGAGTTCCACCAGGTGAGAAGAGTG

>EXON8

AAGCAATTAAAAGAAGTTATAGAGTTGGATGCAAGCGTAACCCGAAGGACAACTGGATGTGTGGAAGCACCAGTTTTCTCCATGTGCTCAGGCTAATCCTCGTTAATACTCAGAACGTTGAGTTTCTACGGAATGGGTTGCATGTCCATACATGTTTTGGTATCTACCCACACACTTTCATGTGGTATGACTGTGCATCCCAGGAGAAAGGCTGTGGTGTGTGTCTCTGCGCCTCAGTGGAGCTGAACAAACGATTGCTGAAAATGGTGTCATAAAGTTCCACCAGGTGAGAAGAGTG

>GFP

AUGGUGAGCA AGGGCGAGGA GCUGUUCACC GGGGUGGUGC CCAUCCUGGU CGAGCUGGACGGCGACGUAA ACGGCCACAA GUUCAGCGUG UCCGGCGAGGGCGAGGGCGA UGCCACCUAC GGCAAGCUGA CCCUGAAGUU CAUCUGCACC ACCGGCAAGC UGCCCGUGCC CUGGCCCACC CUCGUGACCA CCCUGACCUACGGCGUGCAG UGCUUCAGCC GCUACCCCGA CCACAUGAAG CAGCACGACU UCUUCAAGUC CGCCAUGCCC GAAGGCUACG UCCAGGAGCG CACCAUCUUCUUCAAGGACG ACGGCAACUA CAAGACCCGC GCCGAGGUGA AGUUCGAGGG CGACACCCUG GUGAACCGCA UCGAGCUGAA GGGCAUCGAC UUCAAGGAGG ACGGCAACAU CCUGGGGCAC AAGCUGGAGU ACAACUACAA CAGCCACAAC GUCUAUAUCA UGGCCGACAA GCAGAAGAAC GGCAUCAAGG UGAACUUCAA GAUCCGCCAC AACAUCGAGG ACGGCAGCGU GCAGCUCGCC GACCACUACC AGCAGAACAC CCCCAUCGGC GACGGCCCCG UGCUGCUGCC CGACAACCAC UACCUGAGCA CCCAGUCCGC CCUGAGCAAA GACCCCAACG AGAAGCGCGA UCACAUGGUC CUGCUGGAGU UCGUGACCGC CGCCGGGAUC ACUCUCGGCA UGGACGAGCU GUACAAGUAA
