# Supplementary material for: Distinct role of 5′UTR sequences in dendritic trafficking of BDNF mRNA: additional mechanisms for the BDNF splice variants spatial code
Source: Mol Brain. 2021 Jan 12;14:10. doi: 10.1186/s13041-020-00680-8 (PMC7805101; doi:10.1186/s13041-020-00680-8)
Supplement: Supplementary file 4 — Additional file 4. Recognition sequences and p values of the bioinformatic analysis of each 5’UTR BDNF exon using RBPmap database. [file 13041_2020_680_MOESM4_ESM.docx]

Calculation parameters:

Genome: Mouse (mm10)

Selected motifs: All Human/Mouse motifs

Stringency level: High

Conservation filter: On

**********************************************

EXON1

==============================================================================

| Protein: A1CF(Hs/Mm) |  |  |
| --- | --- | --- |
| Sequence Position Motif | K-mer | Z-score P-value |
| 181 wuaauur  Protein: ANKHD1(Hs/Mm) | uuauuug | 2.156 1.55e-02 |
| Sequence Position Motif | K-mer | Z-score P-value |
| 592 agacgww  Protein: CPEB2(Hs/Mm) | agacgag | 1.812 3.50e-02 |
| Sequence Position Motif | K-mer | Z-score P-value |
| 212 chuuuuu | cacuuuu | 3.194 7.02e-04 |
| 214 chuuuuu | cuuuuuc | 3.316 4.57e-04 |
| 227 chuuuuu  Protein: CPEB4(Hs/Mm) | cuuuuug | 3.388 3.52e-04 |
| Sequence Position Motif | K-mer | Z-score P-value |
| 214 uuuuuu | cuuuuu | 3.478 2.53e-04 |
| 227 uuuuuu | cuuuuu | 3.478 2.53e-04 |
| 399 uuuuuu  Protein: CUG-BP(Hs/Mm) | guuuuu | 2.548 5.42e-03 |
| Sequence Position Motif | K-mer | Z-score P-value |
| 218 ugcug | uucug | 1.779 3.76e-02 |
| 234 ugcug | ugcua | 1.743 4.07e-02 |
| 263 ugcug | agcug | 1.743 4.07e-02 |
| 266 ugcug | ugcuu | 1.779 3.76e-02 |
| 421 ugcug | ugcuu | 1.743 4.07e-02 |
| 430 ugcug  Protein: ENOX1(Hs/Mm) | uucug | 1.805 3.55e-02 |
| Sequence Position Motif | K-mer | Z-score P-value |
| 620 hrkacag  Protein: FXR1(Hs/Mm) | aggacag | 1.812 3.50e-02 |
| Sequence Position Motif | K-mer | Z-score P-value |
| 548 aygacr | gaugac | 2.800 2.56e-03 |

| Protein: HNRNPC(Hs/Mm) |  |  |
| --- | --- | --- |
| Sequence Position Motif | K-mer | Z-score P-value |
| 180 huuuuuk | auuauuu | 1.848 3.23e-02 |
| 213 huuuuuk | acuuuuu | 2.982 1.43e-03 |
| 214 huuuuuk | cuuuuuc | 3.089 1.00e-03 |
| 226 huuuuuk | acuuuuu | 2.982 1.43e-03 |
| 227 huuuuuk | cuuuuug | 3.125 8.89e-04 |
| 399 huuuuuk  Protein: HNRNPCL1(Hs/Mm) | guuuuuc | 2.223 1.31e-02 |
| Sequence Position Motif | K-mer | Z-score P-value |
| 180 huuuuuk | auuauuu | 1.825 3.40e-02 |
| 213 huuuuuk | acuuuuu | 2.947 1.60e-03 |
| 214 huuuuuk | cuuuuuc | 3.035 1.20e-03 |
| 226 huuuuuk | acuuuuu | 2.947 1.60e-03 |
| 227 huuuuuk | cuuuuug | 3.070 1.07e-03 |
| 399 huuuuuk  Protein: HNRNPF(Hs/Mm) | guuuuuc | 2.202 1.38e-02 |
| Sequence Position Motif | K-mer | Z-score P-value |
| 446 gugkau  Protein: HNRNPL(Hs/Mm) | guggau | 1.911 2.80e-02 |
| Sequence Position Motif | K-mer | Z-score P-value |
| 562 amayama  Protein: HNRNPM(Hs/Mm) | agacaca | 2.973 1.47e-03 |
| Sequence Position Motif | K-mer | Z-score P-value |
| 418 gguugguu  Protein: HNRPLL(Hs/Mm) | aguugcuu | 2.143 1.61e-02 |
| Sequence Position Motif | K-mer | Z-score P-value |
| 555 rcahaca | ucaaaca | 2.696 3.51e-03 |
| 562 rcahaca  Protein: HuR(Hs/Mm) | agacaca | 2.671 3.78e-03 |
| Sequence Position Motif | K-mer | Z-score P-value |
| 176 uukruuu | uuggauu | 2.143 1.61e-02 |
| 396 uukruuu | uagguuu | 2.187 1.44e-02 |
| 420 uukruuu  Protein: IGF2BP2(Hs/Mm) | uugcuuu | 3.044 1.17e-03 |
| Sequence Position Motif | K-mer | Z-score P-value |
| 555 vmahwca  Protein: MBNL1(Hs/Mm) | ucaaaca | 2.397 8.26e-03 |
| Sequence Position Motif | K-mer | Z-score P-value |
| 212 ygcuky | cacuuu | 1.686 4.59e-02 |
| 242 ygcuky | cgcagu | 2.706 3.41e-03 |

| 263 | ygcuky | agcugc | 2.706 3.41e-03 |
| --- | --- | --- | --- |
| 266 | ygcuky | ugcuuc | 3.118 9.10e-04 |
| 282 | ygcuky | cgcuau | 2.294 1.09e-02 |
| 411 | ygcuky | cgaugc | 2.706 3.41e-03 |
| 421 | ygcuky | ugcuuu | 3.108 9.42e-04 |
| 430  Protein: MSI1(Hs/Mm) | ygcuky | uucugu | 2.657 3.94e-03 |
| Sequence Position | Motif | K-mer | Z-score P-value |
| 289  Protein: NOVA1(Hs/Mm) | uaguwrg | uagcagg | 2.500 6.21e-03 |
| Sequence Position | Motif | K-mer | Z-score P-value |
| 308 | ycay | ucau | 2.750 2.98e-03 |
| 324 | ycay | ucau | 3.380 3.62e-04 |
| 328 | ycay | ucau | 3.380 3.62e-04 |
| 337 | ycay | ucac | 2.750 2.98e-03 |
| 499 | ycay | ucac | 2.750 2.98e-03 |
| 520  Protein: PCBP1(Hs/Mm) | ycay | ccac | 2.750 2.98e-03 |
| Sequence Position | Motif | K-mer | Z-score P-value |
| 57  Protein: PTBP1(Hs/Mm) | ccwwhcc | ccuagcc | 2.909 1.81e-03 |
| Sequence Position | Motif | K-mer | Z-score P-value |
| 78 | ucuu | uuuu | 1.884 2.98e-02 |
| 81 | ucuu | ucuu | 2.580 4.94e-03 |
| 102 | ucuu | ucuc | 2.411 7.95e-03 |
| 104 | ucuu | uccu | 2.375 8.77e-03 |
| 117 | ucuu | ucau | 2.018 2.18e-02 |
| 120 | ucuu | ucuu | 2.580 4.94e-03 |
| 196 | cucucu | uacucu | 2.107 1.76e-02 |
| 198 | cucucu | cucuga | 2.107 1.76e-02 |
| 206 | cucucu | cucccu | 2.670 3.79e-03 |
| 208 | cucucu | cccuca | 2.116 1.72e-02 |
| 210 | cucucu | cucacu | 2.670 3.79e-03 |
| 212 | cucucu | cacuuu | 2.116 1.72e-02 |
| 214 | cucucu | cuuuuu | 2.116 1.72e-02 |
| 216 | cucucu | uuuucu | 2.116 1.72e-02 |
| 227 | cucucu | cuuuuu | 2.107 1.76e-02 |
| 319 | cucucu | cucgcu | 2.107 1.76e-02 |
| 321 | cucucu | cgcuca | 1.741 4.08e-02 |
| 323 | cucucu | cucauu | 1.741 4.08e-02 |
| 361 | cucucu | gugucu | 1.893 2.92e-02 |
| 363 | cucucu | gucucu | 2.420 7.76e-03 |
| 365 | cucucu | cucuca | 2.455 7.04e-03 |
| 422 | ucuu | gcuu | 2.098 1.80e-02 |
| 428 | ucuu | ucuu | 2.446 7.22e-03 |

| 431  Protein: RALY(Hs/Mm) | ucuu | ucug | 1.884 2.98e-02 |
| --- | --- | --- | --- |
| Sequence Position | Motif | K-mer | Z-score P-value |
| 215 | uuuuuub | uuuuucu | 2.982 1.43e-03 |
| 216 | uuuuuub | uuuucug | 3.036 1.20e-03 |
| 227 | uuuuuub | cuuuuug | 3.098 9.74e-04 |
| 228 | uuuuuub | uuuuugu | 2.982 1.43e-03 |
| 229  Protein: RBM24(Hs/Mm) | uuuuuub | uuuugug | 3.036 1.20e-03 |
| Sequence Position | Motif | K-mer | Z-score P-value |
| 137  Protein: RBM41(Hs/Mm) | wgwgugd | gguguga | 1.649 4.96e-02 |
| Sequence Position | Motif | K-mer | Z-score P-value |
| 569  Protein: RBMS1(Hs/Mm) | wuacwuk | uuaccuu | 2.481 6.55e-03 |
| Sequence Position | Motif | K-mer | Z-score P-value |
| 285  Protein: RBMS3(Hs/Mm) | kauauas | uauauag | 2.000 2.27e-02 |
| Sequence Position | Motif | K-mer | Z-score P-value |
| 285  Protein: SRSF1(Hs/Mm) | hauaua | uauaua | 2.000 2.27e-02 |
| Sequence Position | Motif | K-mer | Z-score P-value |
| 42 | crsmsgw | cagcggu | 2.389 8.45e-03 |
| 458 | ugrwgvh | ugaagcu | 2.133 1.65e-02 |
| Protein: SRSF10(Hs/Mm) | |  |  |
| Sequence Position Motif | | K-mer | Z-score P-value |
| 257 agagavm  Protein: SRSF2(Hs/Mm) | | ugagaaa | 2.181 1.46e-02 |
| Sequence Position Motif | | K-mer | Z-score P-value |
| 160 ggagwd | | aggagc | 2.195 1.41e-02 |
| 263 ugcygyy  Protein: SRSF3(Hs/Mm) | | agcugcu | 1.949 2.56e-02 |
| Sequence Position Motif | | K-mer | Z-score P-value |
| 47 cuckucy | | gucuucc | 2.530 5.70e-03 |
| 48 wcwwc | | ucuuc | 2.676 3.73e-03 |
| 52 cuckucy | | cccgccc | 2.290 1.10e-02 |
| 58 cuckucy | | cuagccu | 2.350 9.39e-03 |
| 77 cuckucy | | guuuucu | 1.950 2.56e-02 |
| 80 cuckucy | | uucuuac | 1.910 2.81e-02 |
| 101 cuckucy | | aucuccu | 2.340 9.64e-03 |

| 116 | cuckucy | cucaucu | 2.560 5.23e-03 |
| --- | --- | --- | --- |
| 117 | wcwwc | ucauc | 2.676 3.73e-03 |
| 121 | cuckucy | cuuugcc | 2.200 1.39e-02 |
| 212 | cuckucy | cacuuuu | 1.860 3.14e-02 |
| 214 | cuckucy | cuuuuuc | 1.860 3.14e-02 |
| 215 | cuckucy | uuuuucu | 1.860 3.14e-02 |
| 404 | wcwwc | ucaac | 3.539 2.01e-04 |
| 407 | wcwwc | acauc | 3.539 2.01e-04 |
| 424 | cuckucy | uuugucu | 2.040 2.07e-02 |
| 427 | cuckucy | gucuucu | 2.380 8.66e-03 |
| 428 | wcwwc | ucuuc | 3.167 7.70e-04 |
| 537 | wcwwc | acuuc | 3.294 4.94e-04 |
| 552  Protein: SRSF5(Hs/Mm) | wcwwc | acuuc | 3.382 3.60e-04 |
| Sequence Position | Motif | K-mer | Z-score P-value |
| 49 | yywcwsg | cuucccg | 1.828 3.38e-02 |
| 91 | yywcwsg | cgacagg | 1.925 2.71e-02 |
| 126 | yywcwsg | ccacagc | 2.301 1.07e-02 |
| 132 | yywcwsg | ccccagg | 2.204 1.38e-02 |
| 209 | yywcwsg | ccucacu | 2.710 3.36e-03 |
| 217 | yywcwsg | uuucugg | 3.086 1.01e-03 |
| 247 | yywcwsg | ugacagg | 2.473 6.70e-03 |
| 268 | yywcwsg | cuucagg | 2.140 1.62e-02 |
| 308 | yywcwsg | ucauugg | 1.828 3.38e-02 |
| 349 | yywcwsg | ucaaagg | 2.462 6.91e-03 |
| 364 | yywcwsg | ucucuca | 2.043 2.05e-02 |
| 366 | yywcwsg | ucucaga | 2.043 2.05e-02 |
| 393 | yywcwsg | cuauagg | 1.699 4.47e-02 |
| 499 | yywcwsg | ucacacc | 2.204 1.38e-02 |
| 520 | yywcwsg | ccacuga | 2.548 5.42e-03 |
| 538 | yywcwsg | cuucuca | 2.495 6.30e-03 |
| 540 | yywcwsg | ucucaca | 2.495 6.30e-03 |
| 542 | yywcwsg | ucacaug | 2.495 6.30e-03 |
| 616  Protein: SRSF7(Hs/Mm) | yywcwsg | cuccagg | 1.882 2.99e-02 |
| Sequence Position | Motif | K-mer | Z-score P-value |
| 89 | acgacg | ggcgac | 2.628 4.29e-03 |
| 447 | wggacra | uggauga | 1.957 2.52e-02 |
| 512 | wggacra | ugggcga | 1.957 2.52e-02 |
| 548 | acgacg | gaugac | 2.487 6.44e-03 |
| 593 | acgacg | gacgag | 2.487 6.44e-03 |
| 594 | acgagagay | acgagauuu | 2.550 5.39e-03 |
| Protein: TARDBP(Hs/Mm) | |  |  |
| Sequence Position Motif | | K-mer | Z-score P-value |
| 120 ugugug | | ucuuug | 1.807 3.54e-02 |
| 137 ugugug | | ggugug | 2.294 1.09e-02 |

| 139 | ugugug | ugugac | 1.917 2.76e-02 | |
| --- | --- | --- | --- | --- |
| 151 | ugugug | aguggg | 1.917 2.76e-02 | |
| 166 | ugugug | ggcgug | 2.000 2.27e-02 | |
| 182 | ugugug | uauuug | 1.991 2.32e-02 | |
| 184 | ugugug | uuugua | 1.991 2.32e-02 | |
| 186 | ugugug | uguaug | 2.367 8.97e-03 | |
| 188 | ugugug | uauggg | 1.982 2.37e-02 | |
| 190 | ugugug | uggggg | 1.991 2.32e-02 | |
| 217 | ugugug | uuucug | 1.963 2.48e-02 | |
| 219 | ugugug | ucuggg | 1.963 2.48e-02 | |
| 228 | ugugug | uuuuug | 1.963 2.48e-02 | |
| 230 | ugugug | uuugug | 2.349 9.41e-03 | |
| 232  Protein: TIA1(Hs/Mm) | ugugug | ugugcu | 1.963 2.48e-02 | |
| Sequence Position | Motif | K-mer | Z-score P-value | |
| 215 | uuuuubk | uuuuucu | 3.196 6.97e-04 | |
| 227 | uuuuubk | cuuuuug | 3.196 6.97e-04 | |
| 228 | uuuuubk | uuuuugu | 3.196 6.97e-04 | |
| 400  Protein: TRA2B(Hs/Mm) | uuuuubk | uuuuuca | 1.971 2.44e-02 | |
| Sequence Position | Motif | K-mer | Z-score P-value | |
| 74 | aaguguu | aagguuu | 1.916 2.77e-02 | |
| 75 | aaguguu | agguuuu | 1.916 2.77e-02 | |
| 225 | aaguguu | aacuuuu | 1.821 3.43e-02 | |
| 226 | aaguguu | acuuuuu | 1.684 4.61e-02 | |
| 231 | aaguguu | uugugcu | 1.684 4.61e-02 | |
| 251 | aaguguu | aggcguu | 1.726 4.22e-02 | |
| 359 | aaguguu | acguguc | 1.726 4.22e-02 | |
| 377 | aaguguu | gggcguu | 1.705 4.41e-02 | |
| 397 | aaguguu | agguuuu | 1.821 3.43e-02 | |
| 460 | aaguguu | aagcuug | 1.916 2.77e-02 | |
| 477 | aaguguu | aaagggu | 1.916 2.77e-02 | |
| 478 | aaguguu | aaggguu | 2.295 1.09e-02 | |
| 496 | aaguguu | aagucac | 1.916 2.77e-02 | |
| 635  Protein: U2AF2(Hs/Mm) | aaguguu | caauguu | 1.916 2.77e-02 | |
| Sequence Position | Motif | K-mer | Z-score P-value | |
| 214 | uuuuuyc | cuuuuuc | 3.162 7.83e-04 | |
| 215 | uuuuuyc | uuuuucu | 3.057 1.12e-03 | |
| 399 | uuuuuyc | guuuuuc | 2.305 1.06e-02 | |
| 400  Protein: YBX1(Hs/Mm) | uuuuuyc | uuuuuca | 2.181 1.46e-02 | |
| Sequence Position | Motif | K-mer | Z-score P-value | |
| 578 | aacaucd | agcaucu | 2.273 1.15e-02 | |
| Protein: ZC3H14(Hs/Mm) | |  |  |  |
| Sequence Position Motif | | K-mer | Z-score P-value |  |
| 184 uuuduuu | | uuuguau | 1.979 2.39e-02 |  |
| 424 uuuduuu | | uuugucu | 2.361 9.11e-03 |  |

************************************************************************************************

EXON2A

==============================================================================

| Protein: A1CF(Hs/Mm) |  |  |
| --- | --- | --- |
| Sequence Position Motif | K-mer | Z-score P-value |
| 77 wuaauur  Protein: BRUNOL4(Hs/Mm) | uuaauua | 1.878 3.02e-02 |
| Sequence Position Motif | K-mer | Z-score P-value |
| 121 kgugukk | ggugugu | 2.770 2.80e-03 |
| 123 kgugukk  Protein: BRUNOL5(Hs/Mm) | uguguga | 2.770 2.80e-03 |
| Sequence Position Motif | K-mer | Z-score P-value |
| 121 ugugukk | ggugugu | 2.800 2.56e-03 |
| 123 ugugukk  Protein: CPEB2(Hs/Mm) | uguguga | 2.825 2.36e-03 |
| Sequence Position Motif | K-mer | Z-score P-value |
| 89 chuuuuu | uauuuuu | 3.133 8.65e-04 |
| 90 chuuuuu | auuuuuu | 3.194 7.02e-04 |
| 91 chuuuuu  Protein: CPEB4(Hs/Mm) | uuuuuuu | 3.224 6.32e-04 |
| Sequence Position Motif | K-mer | Z-score P-value |
| 90 uuuuuu | auuuuu | 3.357 3.94e-04 |
| 91 uuuuuu | uuuuuu | 3.383 3.58e-04 |
| 92 uuuuuu  Protein: CUG-BP(Hs/Mm) | uuuuuu | 3.383 3.58e-04 |
| Sequence Position Motif | K-mer | Z-score P-value |
| 108 ugcug | uccug | 2.248 1.23e-02 |
| 111 ugcug  Protein: HNRNPA1(Hs/Mm) | ugcug | 2.690 3.57e-03 |
| Sequence Position Motif | K-mer | Z-score P-value |
| 187 guaguagu | gugguagu | 4.098 2.08e-05 |
| 190 guaguagu  Protein: HNRNPA2B1(Hs/Mm) | guaguacu | 4.016 2.96e-05 |
| Sequence Position Motif | K-mer | Z-score P-value |
| 37 gguaguag | ggaagugg | 2.492 6.35e-03 |

| 186 gguaguag | ggugguag | 2.369 8.92e-03 |
| --- | --- | --- |
| 189 gguaguag  Protein: HNRNPC(Hs/Mm) | gguaguac | 2.400 8.20e-03 |
| Sequence Position Motif | K-mer | Z-score P-value |
| 88 huuuuuk | auauuuu | 3.241 5.96e-04 |
| 90 huuuuuk | auuuuuu | 3.652 1.30e-04 |
| 91 huuuuuk | uuuuuuu | 3.527 2.10e-04 |
| 92 huuuuuk  Protein: HNRNPCL1(Hs/Mm) | uuuuuua | 3.304 4.77e-04 |
| Sequence Position Motif | K-mer | Z-score P-value |
| 88 huuuuuk | auauuuu | 3.219 6.43e-04 |
| 90 huuuuuk | auuuuuu | 3.640 1.36e-04 |
| 91 huuuuuk | uuuuuuu | 3.535 2.04e-04 |
| 92 huuuuuk  Protein: HNRNPF(Hs/Mm) | uuuuuua | 3.298 4.87e-04 |
| Sequence Position Motif | K-mer | Z-score P-value |
| 120 gggug | gggug | 2.955 1.56e-03 |
| 122 gggug | gugug | 2.509 6.05e-03 |
| 124 gggug | gugug | 2.509 6.05e-03 |
| 133 gggug  Protein: HuR(Hs/Mm) | gggcg | 2.509 6.05e-03 |
| Sequence Position Motif | K-mer | Z-score P-value |
| 91 uukruuu  Protein: MBNL1(Hs/Mm) | uuuuuuu | 2.681 3.67e-03 |
| Sequence Position Motif | K-mer | Z-score P-value |
| 108 ygcuky | uccugc | 2.500 6.21e-03 |
| 111 ygcuky  Protein: PABPC3(Hs/Mm) | ugcugc | 2.912 1.80e-03 |
| Sequence Position Motif | K-mer | Z-score P-value |
| 27 raaaacm  Protein: PCBP3(Hs/Mm) | caaaacg | 2.898 1.88e-03 |
| Sequence Position Motif | K-mer | Z-score P-value |
| 102 uuuycc  Protein: RALY(Hs/Mm) | uuuucc | 1.703 4.43e-02 |
| Sequence Position Motif | K-mer | Z-score P-value |
| 90 uuuuuub | auuuuuu | 3.116 9.17e-04 |
| 91 uuuuuub | uuuuuuu | 3.438 2.93e-04 |
| 92 uuuuuub  Protein: RBM24(Hs/Mm) | uuuuuua | 3.393 3.46e-04 |
| Sequence Position Motif | K-mer | Z-score P-value |

| 121 wgwgugd | | ggugugu | 2.987 1.41e-03 |
| --- | --- | --- | --- |
| 123 wgwgugd  Protein: RBM38(Hs/Mm) | | uguguga | 3.260 5.57e-04 |
| Sequence Position Motif | | K-mer | Z-score P-value |
| 120 kkguguk | | gggugug | 3.321 4.48e-04 |
| 122 kkguguk  Protein: RBM6(Hs/Mm) | | gugugug | 3.513 2.22e-04 |
| Sequence Position Motif | | K-mer | Z-score P-value |
| 199 hauccar  Protein: SNRNP70(Hs/Mm) | | cauccag | 2.085 1.85e-02 |
| Sequence Position Motif | | K-mer | Z-score P-value |
| 64 rwucaag  Protein: SRSF10(Hs/Mm) | | uaucaag | 1.877 3.03e-02 |
| Sequence Position | Motif | K-mer | Z-score P-value |
| 57  Protein: SRSF2(Hs/Mm) | agagavm | agagcaa | 2.108 1.75e-02 |
| Sequence Position | Motif | K-mer | Z-score P-value |
| 108 | ugcygyy | uccugcu | 2.313 1.04e-02 |
| 111  Protein: SRSF3(Hs/Mm) | ugcygyy | ugcugcg | 2.313 1.04e-02 |
| Sequence Position | Motif | K-mer | Z-score P-value |
| 101 | cuckucy | cuuuucc | 2.580 4.94e-03 |
| 104 | cuckucy | uuccucc | 2.220 1.32e-02 |
| 107 | cuckucy | cuccugc | 2.220 1.32e-02 |
| 195 | wcwwc | acuuc | 3.167 7.70e-04 |
| 197 | cuckucy | uucaucc | 1.820 3.44e-02 |
| 198  Protein: SRSF7(Hs/Mm) | wcwwc | ucauc | 3.167 7.70e-04 |
| Sequence Position | Motif | K-mer | Z-score P-value |
| 42 | wggacra | uggaaga | 2.436 7.43e-03 |
| Protein: TARDBP(Hs/Mm) | |  |  |
| Sequence Position Motif | | K-mer | Z-score P-value |
| 119 ugugug | | cgggug | 2.486 6.46e-03 |
| 121 ugugug | | ggugug | 3.055 1.13e-03 |
| 123 ugugug | | ugugug | 3.440 2.91e-04 |
| 125 ugugug  Protein: TIA1(Hs/Mm) | | ugugau | 2.486 6.46e-03 |
| Sequence Position Motif | | K-mer | Z-score P-value |
| 90 uuuuubk | | auuuuuu | 3.176 7.47e-04 |
| 91 uuuuubk | | uuuuuuu | 3.451 2.79e-04 |
| 92 uuuuubk  Protein: U2AF2(Hs/Mm) | | uuuuuua | 3.382 3.60e-04 |
| Sequence Position Motif | | K-mer | Z-score P-value |
| 91 uuuuuyc | | uuuuuuu | 3.352 4.01e-04 |
| 92 uuuuuyc | | uuuuuua | 3.314 4.60e-04 |
| 101 uuuuuyc  Protein: ZC3H10(Hs/Mm) | | cuuuucc | 3.200 6.87e-04 |
| Sequence Position Motif | | K-mer | Z-score P-value |
| 169 ssagcgm  Protein: ZC3H14(Hs/Mm) | | ccagcgg | 2.397 8.26e-03 |
| Sequence Position Motif | | K-mer | Z-score P-value |
| 91 uuuduuu  Protein: ZCRB1(Hs/Mm) | | uuuuuuu | 2.856 2.15e-03 |
| Sequence Position Motif | | K-mer | Z-score P-value |
| 74 grhuuaa | | cacuuaa | 2.488 6.42e-03 |

************************************************************************************************

EXON2B

==============================================================================

| Protein: A1CF(Hs/Mm) |  |  |
| --- | --- | --- |
| Sequence Position Motif | K-mer | Z-score P-value |
| 77 wuaauur  Protein: BRUNOL4(Hs/Mm) | uuaauua | 1.878 3.02e-02 |
| Sequence Position Motif | K-mer | Z-score P-value |
| 121 kgugukk | ggugugu | 2.770 2.80e-03 |
| 123 kgugukk  Protein: BRUNOL5(Hs/Mm) | uguguga | 2.770 2.80e-03 |
| Sequence Position Motif | K-mer | Z-score P-value |
| 121 ugugukk | ggugugu | 2.800 2.56e-03 |
| 123 ugugukk  Protein: CNOT4(Hs/Mm) | uguguga | 2.825 2.36e-03 |
| Sequence Position Motif | K-mer | Z-score P-value |
| 294 gacaga | gagaga | 3.163 7.81e-04 |
| 296 gacaga  Protein: CPEB2(Hs/Mm) | gagaga | 3.163 7.81e-04 |
| Sequence Position Motif | K-mer | Z-score P-value |
| 89 chuuuuu | uauuuuu | 3.133 8.65e-04 |
| 90 chuuuuu | auuuuuu | 3.194 7.02e-04 |
| 91 chuuuuu | uuuuuuu | 3.224 6.32e-04 |

| Protein: CPEB4(Hs/Mm) |  |  |
| --- | --- | --- |
| Sequence Position Motif | K-mer | Z-score P-value |
| 90 uuuuuu | auuuuu | 3.357 3.94e-04 |
| 91 uuuuuu | uuuuuu | 3.383 3.58e-04 |
| 92 uuuuuu  Protein: CUG-BP(Hs/Mm) | uuuuuu | 3.383 3.58e-04 |
| Sequence Position Motif | K-mer | Z-score P-value |
| 108 ugcug | uccug | 2.248 1.23e-02 |
| 111 ugcug  Protein: HNRNPA1(Hs/Mm) | ugcug | 2.690 3.57e-03 |
| Sequence Position Motif | K-mer | Z-score P-value |
| 187 guaguagu | gugguagu | 4.098 2.08e-05 |
| 190 guaguagu  Protein: HNRNPA2B1(Hs/Mm) | guaguacu | 4.016 2.96e-05 |
| Sequence Position Motif | K-mer | Z-score P-value |
| 37 gguaguag | ggaagugg | 2.492 6.35e-03 |
| 186 gguaguag | ggugguag | 2.369 8.92e-03 |
| 189 gguaguag  Protein: HNRNPC(Hs/Mm) | gguaguac | 2.400 8.20e-03 |
| Sequence Position Motif | K-mer | Z-score P-value |
| 88 huuuuuk | auauuuu | 3.241 5.96e-04 |
| 90 huuuuuk | auuuuuu | 3.652 1.30e-04 |
| 91 huuuuuk | uuuuuuu | 3.527 2.10e-04 |
| 92 huuuuuk | uuuuuua | 3.304 4.77e-04 |
| 208 huuuuuk  Protein: HNRNPCL1(Hs/Mm) | auucuuu | 2.188 1.43e-02 |
| Sequence Position Motif | K-mer | Z-score P-value |
| 88 huuuuuk | auauuuu | 3.219 6.43e-04 |
| 90 huuuuuk | auuuuuu | 3.640 1.36e-04 |
| 91 huuuuuk | uuuuuuu | 3.535 2.04e-04 |
| 92 huuuuuk | uuuuuua | 3.298 4.87e-04 |
| 208 huuuuuk  Protein: HNRNPF(Hs/Mm) | auucuuu | 2.184 1.45e-02 |
| Sequence Position Motif | K-mer | Z-score P-value |
| 120 gggug | gggug | 2.955 1.56e-03 |
| 122 gggug | gugug | 2.509 6.05e-03 |
| 124 gggug | gugug | 2.509 6.05e-03 |
| 133 gggug  Protein: HNRNPK(Hs/Mm) | gggcg | 2.509 6.05e-03 |
| Sequence Position Motif | K-mer | Z-score P-value |
| 229 ccawmcc | ccaaccc | 2.072 1.91e-02 |

| Protein: HNRNPM(Hs/Mm) |  |  |
| --- | --- | --- |
| Sequence Position Motif | K-mer | Z-score P-value |
| 341 gguugguu  Protein: HuR(Hs/Mm) | guuugguc | 2.190 1.43e-02 |
| Sequence Position Motif | K-mer | Z-score P-value |
| 91 uukruuu  Protein: KHDRBS1(Hs/Mm) | uuuuuuu | 2.681 3.67e-03 |
| Sequence Position Motif | K-mer | Z-score P-value |
| 246 auaaaav  Protein: LIN28A(Hs/Mm) | uuaaaaa | 2.072 1.91e-02 |
| Sequence Position Motif | K-mer | Z-score P-value |
| 292 hggagwa | uggagag | 2.757 2.92e-03 |
| 325 hggagwa  Protein: MBNL1(Hs/Mm) | uggagag | 2.284 1.12e-02 |
| Sequence Position Motif | K-mer | Z-score P-value |
| 108 ygcuky | uccugc | 2.500 6.21e-03 |
| 111 ygcuky | ugcugc | 2.912 1.80e-03 |
| 194 ygcuky | uacuuc | 2.706 3.41e-03 |
| 209 ygcuky | uucuuu | 2.686 3.62e-03 |
| 219 ygcuky | cgcugu | 3.216 6.50e-04 |
| 235 ygcuky | cggugu | 2.500 6.21e-03 |
| 362 ygcuky | cgcuga | 2.294 1.09e-02 |
| 372 ygcuky | ggcuuc | 2.284 1.12e-02 |
| 382 ygcuky  Protein: PABPC3(Hs/Mm) | cggugu | 2.275 1.15e-02 |
| Sequence Position Motif | K-mer | Z-score P-value |
| 27 raaaacm  Protein: PCBP3(Hs/Mm) | caaaacg | 2.898 1.88e-03 |
| Sequence Position Motif | K-mer | Z-score P-value |
| 102 uuuycc | uuuucc | 1.703 4.43e-02 |
| 212 uuuycc | uuuucc | 1.703 4.43e-02 |
| 258 uuuycc  Protein: PTBP1(Hs/Mm) | uuuucc | 1.703 4.43e-02 |
| Sequence Position Motif | K-mer | Z-score P-value |
| 195 ucuu | acuu | 2.134 1.64e-02 |
| 198 ucuu | ucau | 2.134 1.64e-02 |
| 207 ucuu | uauu | 2.134 1.64e-02 |
| 209 cucucu | uucuuu | 1.839 3.30e-02 |
| 210 ucuu | ucuu | 2.688 3.59e-03 |
| 212 ucuu | uuuu | 2.134 1.64e-02 |
| 215 ucuu | uccu | 2.134 1.64e-02 |

| 217 | cucucu | cucgcu | 2.259 1.19e-02 |
| --- | --- | --- | --- |
| 219 | cucucu | cgcugu | 1.884 2.98e-02 |
| 221 | cucucu | cuguca | 1.884 2.98e-02 |
| 241 | cucucu | cgcccu | 1.884 2.98e-02 |
| 244 | ucuu | ccuu | 1.884 2.98e-02 |
| 256 | ucuu | ucuu | 2.446 7.22e-03 |
| 258 | ucuu | uuuu | 1.884 2.98e-02 |
| 349 | cucucu | cccuca | 1.839 3.30e-02 |
| 351 | cucucu | cucauu | 1.839 3.30e-02 |
| 360 | cucucu | cucgcu | 2.214 1.34e-02 |
| 374  Protein: RALY(Hs/Mm) | cucucu | cuuccu | 1.884 2.98e-02 |
| Sequence Position | Motif | K-mer | Z-score P-value |
| 90 | uuuuuub | auuuuuu | 3.116 9.17e-04 |
| 91 | uuuuuub | uuuuuuu | 3.438 2.93e-04 |
| 92  Protein: RBM24(Hs/Mm) | uuuuuub | uuuuuua | 3.393 3.46e-04 |
| Sequence Position | Motif | K-mer | Z-score P-value |
| 121 | wgwgugd | ggugugu | 2.987 1.41e-03 |
| 123  Protein: RBM28(Hs/Mm) | wgwgugd | uguguga | 3.260 5.57e-04 |
| Sequence Position | Motif | K-mer | Z-score P-value |
| 384  Protein: RBM38(Hs/Mm) | gwguagd | guguagg | 2.042 2.06e-02 |
| Sequence Position | Motif | K-mer | Z-score P-value |
| 120 | kkguguk | gggugug | 3.321 4.48e-04 |
| 122 | kkguguk | gugugug | 3.513 2.22e-04 |
| Protein: SNRNP70(Hs/Mm) | |  |  |
| Sequence Position Motif | | K-mer | Z-score P-value |
| 64 rwucaag  Protein: SRSF10(Hs/Mm) | | uaucaag | 1.877 3.03e-02 |
| Sequence Position Motif | | K-mer | Z-score P-value |
| 57 agagavm | | agagcaa | 2.108 1.75e-02 |
| 295 agagavm  Protein: SRSF2(Hs/Mm) | | agagaga | 3.241 5.96e-04 |
| Sequence Position Motif | | K-mer | Z-score P-value |
| 108 ugcygyy | | uccugcu | 2.313 1.04e-02 |
| 111 ugcygyy | | ugcugcg | 2.313 1.04e-02 |
| 219 ugcygyy | | cgcuguc | 2.535 5.62e-03 |
| 238 ugcygyy | | ugucgcc | 2.313 1.04e-02 |
| 292 ggagwd | | uggaga | 2.322 1.01e-02 |
| 325 ggagwd | | uggaga | 2.322 1.01e-02 |

| Protein: SRSF3(Hs/Mm) |  |  |  |
| --- | --- | --- | --- |
| Sequence Position | Motif | K-mer | Z-score P-value |
| 101 | cuckucy | cuuuucc | 2.580 4.94e-03 |
| 104 | cuckucy | uuccucc | 2.220 1.32e-02 |
| 107 | cuckucy | cuccugc | 2.220 1.32e-02 |
| 195 | wcwwc | acuuc | 3.353 4.00e-04 |
| 197 | cuckucy | uucaucc | 2.350 9.39e-03 |
| 198 | wcwwc | ucauc | 3.353 4.00e-04 |
| 206 | cuckucy | guauucu | 2.310 1.04e-02 |
| 209 | cuckucy | uucuuuu | 2.310 1.04e-02 |
| 211 | cuckucy | cuuuucc | 2.670 3.79e-03 |
| 239 | cuckucy | gucgccc | 2.300 1.07e-02 |
| 252 | cuckucy | agcgucu | 2.330 9.90e-03 |
| 255 | cuckucy | gucuuuu | 2.330 9.90e-03 |
| 257 | cuckucy | cuuuucc | 2.690 3.57e-03 |
| 268 | cuckucy | uucggcu | 2.270 1.16e-02 |
| 401  Protein: SRSF5(Hs/Mm) | cuckucy | cucuugg | 1.670 4.75e-02 |
| Sequence Position | Motif | K-mer | Z-score P-value |
| 214 | yywcwsg | uuccucg | 1.914 2.78e-02 |
| 274  Protein: SRSF7(Hs/Mm) | yywcwsg | ucacacu | 1.882 2.99e-02 |
| Sequence Position | Motif | K-mer | Z-score P-value |
| 42 | wggacra | uggaaga | 2.436 7.43e-03 |
| Protein: TARDBP(Hs/Mm) | |  |  |
| Sequence Position Motif | | K-mer | Z-score P-value |
| 119 ugugug | | cgggug | 2.486 6.46e-03 |
| 121 ugugug | | ggugug | 3.055 1.13e-03 |
| 123 ugugug | | ugugug | 3.440 2.91e-04 |
| 125 ugugug  Protein: TIA1(Hs/Mm) | | ugugau | 2.486 6.46e-03 |
| Sequence Position Motif | | K-mer | Z-score P-value |
| 90 uuuuubk | | auuuuuu | 3.176 7.47e-04 |
| 91 uuuuubk | | uuuuuuu | 3.451 2.79e-04 |
| 92 uuuuubk  Protein: TRA2B(Hs/Mm) | | uuuuuua | 3.382 3.60e-04 |
| Sequence Position Motif | | K-mer | Z-score P-value |
| 185 aaguguu | | agguggu | 2.105 1.76e-02 |
| 204 aaguguu | | agguauu | 1.916 2.77e-02 |
| 318 aaguguu  Protein: U2AF2(Hs/Mm) | | gagcguu | 1.726 4.22e-02 |
| Sequence Position Motif | | K-mer | Z-score P-value |
| 91 uuuuuyc | | uuuuuuu | 3.352 4.01e-04 |
| 92 uuuuuyc | | uuuuuua | 3.314 4.60e-04 |
| 101 uuuuuyc | | cuuuucc | 3.200 6.87e-04 |
| 210 uuuuuyc | | ucuuuuc | 2.629 4.28e-03 |
| 211 uuuuuyc | | cuuuucc | 2.657 3.94e-03 |
| 256 uuuuuyc | | ucuuuuc | 2.048 2.03e-02 |
| 257 uuuuuyc  Protein: ZC3H10(Hs/Mm) | | cuuuucc | 2.076 1.89e-02 |
| Sequence Position Motif | | K-mer | Z-score P-value |
| 169 ssagcgm  Protein: ZC3H14(Hs/Mm) | | ccagcgg | 2.397 8.26e-03 |
| Sequence Position Motif | | K-mer | Z-score P-value |
| 91 uuuduuu  Protein: ZCRB1(Hs/Mm) | | uuuuuuu | 2.856 2.15e-03 |
| Sequence Position Motif | | K-mer | Z-score P-value |
| 74 grhuuaa | | cacuuaa | 2.488 6.42e-03 |

************************************************************************************************

EXON2C

==============================================================================

| Protein: A1CF(Hs/Mm) |  |  |
| --- | --- | --- |
| Sequence Position Motif | K-mer | Z-score P-value |
| 77 wuaauur  Protein: ANKHD1(Hs/Mm) | uuaauua | 1.878 3.02e-02 |
| Sequence Position Motif | K-mer | Z-score P-value |
| 496 agacgww  Protein: BRUNOL4(Hs/Mm) | agaaguu | 2.623 4.36e-03 |
| Sequence Position Motif | K-mer | Z-score P-value |
| 121 kgugukk | ggugugu | 2.770 2.80e-03 |
| 123 kgugukk  Protein: BRUNOL5(Hs/Mm) | uguguga | 2.770 2.80e-03 |
| Sequence Position Motif | K-mer | Z-score P-value |
| 121 ugugukk | ggugugu | 2.800 2.56e-03 |
| 123 ugugukk  Protein: CNOT4(Hs/Mm) | uguguga | 2.825 2.36e-03 |
| Sequence Position Motif | K-mer | Z-score P-value |
| 294 gacaga | gagaga | 3.163 7.81e-04 |
| 296 gacaga | gagaga | 3.163 7.81e-04 |

Protein: CPEB2(Hs/Mm)

| Sequence Position Motif | K-mer | Z-score P-value |
| --- | --- | --- |
| 89 chuuuuu | uauuuuu | 3.133 8.65e-04 |
| 90 chuuuuu | auuuuuu | 3.194 7.02e-04 |
| 91 chuuuuu  Protein: CPEB4(Hs/Mm) | uuuuuuu | 3.224 6.32e-04 |
| Sequence Position Motif | K-mer | Z-score P-value |
| 90 uuuuuu | auuuuu | 3.357 3.94e-04 |
| 91 uuuuuu | uuuuuu | 3.383 3.58e-04 |
| 92 uuuuuu  Protein: CUG-BP(Hs/Mm) | uuuuuu | 3.383 3.58e-04 |
| Sequence Position Motif | K-mer | Z-score P-value |
| 108 ugcug | uccug | 2.248 1.23e-02 |
| 111 ugcug  Protein: ESRP2(Hs/Mm) | ugcug | 2.690 3.57e-03 |
| Sequence Position Motif | K-mer | Z-score P-value |
| 439 ugggrad  Protein: HNRNPA1(Hs/Mm) | ugggaaa | 2.355 9.26e-03 |
| Sequence Position Motif | K-mer | Z-score P-value |
| 187 guaguagu | gugguagu | 4.098 2.08e-05 |
| 190 guaguagu  Protein: HNRNPA2B1(Hs/Mm) | guaguacu | 4.016 2.96e-05 |
| Sequence Position Motif | K-mer | Z-score P-value |
| 37 gguaguag | ggaagugg | 2.492 6.35e-03 |
| 186 gguaguag | ggugguag | 2.369 8.92e-03 |
| 189 gguaguag  Protein: HNRNPC(Hs/Mm) | gguaguac | 2.400 8.20e-03 |
| Sequence Position Motif | K-mer | Z-score P-value |
| 88 huuuuuk | auauuuu | 3.241 5.96e-04 |
| 90 huuuuuk | auuuuuu | 3.652 1.30e-04 |
| 91 huuuuuk | uuuuuuu | 3.527 2.10e-04 |
| 92 huuuuuk | uuuuuua | 3.304 4.77e-04 |
| 208 huuuuuk  Protein: HNRNPCL1(Hs/Mm) | auucuuu | 2.188 1.43e-02 |
| Sequence Position Motif | K-mer | Z-score P-value |
| 88 huuuuuk | auauuuu | 3.219 6.43e-04 |
| 90 huuuuuk | auuuuuu | 3.640 1.36e-04 |
| 91 huuuuuk | uuuuuuu | 3.535 2.04e-04 |
| 92 huuuuuk | uuuuuua | 3.298 4.87e-04 |
| 208 huuuuuk  Protein: HNRNPF(Hs/Mm) | auucuuu | 2.184 1.45e-02 |
| Sequence Position Motif | K-mer | Z-score P-value |

| 120 gggug | gggug | 2.955 1.56e-03 |
| --- | --- | --- |
| 122 gggug | gugug | 2.509 6.05e-03 |
| 124 gggug | gugug | 2.509 6.05e-03 |
| 133 gggug  Protein: HNRNPK(Hs/Mm) | gggcg | 2.509 6.05e-03 |
| Sequence Position Motif | K-mer | Z-score P-value |
| 229 ccawmcc  Protein: HNRNPM(Hs/Mm) | ccaaccc | 2.072 1.91e-02 |
| Sequence Position Motif | K-mer | Z-score P-value |
| 341 gguugguu | guuugguc | 2.190 1.43e-02 |
| 417 gguugguu  Protein: HuR(Hs/Mm) | gguuggua | 1.810 3.51e-02 |
| Sequence Position Motif | K-mer | Z-score P-value |
| 91 uukruuu | uuuuuuu | 2.681 3.67e-03 |
| 419 uukruuu  Protein: KHDRBS1(Hs/Mm) | uugguau | 1.868 3.09e-02 |
| Sequence Position Motif | K-mer | Z-score P-value |
| 246 auaaaav  Protein: LIN28A(Hs/Mm) | uuaaaaa | 2.072 1.91e-02 |
| Sequence Position Motif | K-mer | Z-score P-value |
| 292 hggagwa | uggagag | 2.757 2.92e-03 |
| 325 hggagwa  Protein: MBNL1(Hs/Mm) | uggagag | 2.284 1.12e-02 |
| Sequence Position Motif | K-mer | Z-score P-value |
| 108 ygcuky | uccugc | 2.500 6.21e-03 |
| 111 ygcuky | ugcugc | 2.912 1.80e-03 |
| 194 ygcuky | uacuuc | 2.706 3.41e-03 |
| 209 ygcuky | uucuuu | 2.686 3.62e-03 |
| 219 ygcuky | cgcugu | 3.216 6.50e-04 |
| 235 ygcuky | cggugu | 2.500 6.21e-03 |
| 362 ygcuky | cgcuga | 2.294 1.09e-02 |
| 372 ygcuky | ggcuuc | 2.284 1.12e-02 |
| 382 ygcuky  Protein: PABPC3(Hs/Mm) | cggugu | 2.275 1.15e-02 |
| Sequence Position Motif | K-mer | Z-score P-value |
| 27 raaaacm  Protein: PCBP3(Hs/Mm) | caaaacg | 2.898 1.88e-03 |
| Sequence Position Motif | K-mer | Z-score P-value |
| 102 uuuycc | uuuucc | 1.703 4.43e-02 |
| 212 uuuycc | uuuucc | 1.703 4.43e-02 |
| 258 uuuycc | uuuucc | 1.703 4.43e-02 |

| Protein: PTBP1(Hs/Mm) |  |  |  |
| --- | --- | --- | --- |
| Sequence Position | Motif | K-mer | Z-score P-value |
| 195 | ucuu | acuu | 2.134 1.64e-02 |
| 198 | ucuu | ucau | 2.134 1.64e-02 |
| 207 | ucuu | uauu | 2.134 1.64e-02 |
| 209 | cucucu | uucuuu | 1.839 3.30e-02 |
| 210 | ucuu | ucuu | 2.688 3.59e-03 |
| 212 | ucuu | uuuu | 2.134 1.64e-02 |
| 215 | ucuu | uccu | 2.134 1.64e-02 |
| 217 | cucucu | cucgcu | 2.259 1.19e-02 |
| 219 | cucucu | cgcugu | 1.884 2.98e-02 |
| 221 | cucucu | cuguca | 1.884 2.98e-02 |
| 241 | cucucu | cgcccu | 1.884 2.98e-02 |
| 244 | ucuu | ccuu | 1.884 2.98e-02 |
| 256 | ucuu | ucuu | 2.446 7.22e-03 |
| 258 | ucuu | uuuu | 1.884 2.98e-02 |
| 349 | cucucu | cccuca | 1.839 3.30e-02 |
| 351 | cucucu | cucauu | 1.839 3.30e-02 |
| 360 | cucucu | cucgcu | 2.214 1.34e-02 |
| 374  Protein: RALY(Hs/Mm) | cucucu | cuuccu | 1.884 2.98e-02 |
| Sequence Position | Motif | K-mer | Z-score P-value |
| 90 | uuuuuub | auuuuuu | 3.116 9.17e-04 |
| 91 | uuuuuub | uuuuuuu | 3.438 2.93e-04 |
| 92  Protein: RBM24(Hs/Mm) | uuuuuub | uuuuuua | 3.393 3.46e-04 |
| Sequence Position | Motif | K-mer | Z-score P-value |
| 121 | wgwgugd | ggugugu | 2.987 1.41e-03 |
| 123  Protein: RBM28(Hs/Mm) | wgwgugd | uguguga | 3.260 5.57e-04 |
| Sequence Position | Motif | K-mer | Z-score P-value |
| 384  Protein: RBM38(Hs/Mm) | gwguagd | guguagg | 2.042 2.06e-02 |
| Sequence Position | Motif | K-mer | Z-score P-value |
| 120 | kkguguk | gggugug | 3.321 4.48e-04 |
| 122  Protein: SFPQ(Hs/Mm) | kkguguk | gugugug | 3.513 2.22e-04 |
| Sequence Position | Motif | K-mer | Z-score P-value |
| 480 | kurrukk | guggugu | 1.685 4.60e-02 |
| Protein: SNRNP70(Hs/Mm) | |  |  |
| Sequence Position Motif | | K-mer | Z-score P-value |
| 64 rwucaag | | uaucaag | 1.877 3.03e-02 |

| Protein: SRSF10(Hs/Mm) | |  |  |
| --- | --- | --- | --- |
| Sequence Position | Motif | K-mer | Z-score P-value |
| 57 | agagavm | agagcaa | 2.108 1.75e-02 |
| 295  Protein: SRSF2(Hs/Mm) | agagavm | agagaga | 3.241 5.96e-04 |
| Sequence Position | Motif | K-mer | Z-score P-value |
| 108 | ugcygyy | uccugcu | 2.313 1.04e-02 |
| 111 | ugcygyy | ugcugcg | 2.313 1.04e-02 |
| 219 | ugcygyy | cgcuguc | 2.535 5.62e-03 |
| 238 | ugcygyy | ugucgcc | 2.313 1.04e-02 |
| 292 | ggagwd | uggaga | 2.322 1.01e-02 |
| 325  Protein: SRSF3(Hs/Mm) | ggagwd | uggaga | 2.322 1.01e-02 |
| Sequence Position | Motif | K-mer | Z-score P-value |
| 101 | cuckucy | cuuuucc | 2.580 4.94e-03 |
| 104 | cuckucy | uuccucc | 2.220 1.32e-02 |
| 107 | cuckucy | cuccugc | 2.220 1.32e-02 |
| 195 | wcwwc | acuuc | 3.353 4.00e-04 |
| 197 | cuckucy | uucaucc | 2.350 9.39e-03 |
| 198 | wcwwc | ucauc | 3.353 4.00e-04 |
| 206 | cuckucy | guauucu | 2.310 1.04e-02 |
| 209 | cuckucy | uucuuuu | 2.310 1.04e-02 |
| 211 | cuckucy | cuuuucc | 2.670 3.79e-03 |
| 239 | cuckucy | gucgccc | 2.300 1.07e-02 |
| 252 | cuckucy | agcgucu | 2.330 9.90e-03 |
| 255 | cuckucy | gucuuuu | 2.330 9.90e-03 |
| 257 | cuckucy | cuuuucc | 2.690 3.57e-03 |
| 268  Protein: SRSF5(Hs/Mm) | cuckucy | uucggcu | 2.270 1.16e-02 |
| Sequence Position | Motif | K-mer | Z-score P-value |
| 214 | yywcwsg | uuccucg | 1.914 2.78e-02 |
| 274 | yywcwsg | ucacacu | 1.882 2.99e-02 |
| 424 | yywcwsg | auacugg | 1.882 2.99e-02 |
| 459 | yywcwsg | cuccagg | 2.269 1.16e-02 |
| 472  Protein: SRSF7(Hs/Mm) | yywcwsg | ccaccgg | 2.086 1.85e-02 |
| Sequence Position | Motif | K-mer | Z-score P-value |
| 42 | wggacra | uggaaga | 2.436 7.43e-03 |
| Protein: TARDBP(Hs/Mm) | |  |  |
| Sequence Position Motif | | K-mer | Z-score P-value |
| 119 ugugug | | cgggug | 2.486 6.46e-03 |
| 121 ugugug | | ggugug | 3.055 1.13e-03 |
| 123 ugugug | | ugugug | 3.440 2.91e-04 |
| 125  Protein: TIA1(Hs/Mm) | ugugug | ugugau | 2.486 6.46e-03 |
| Sequence Position | Motif | K-mer | Z-score P-value |
| 90 | uuuuubk | auuuuuu | 3.176 7.47e-04 |
| 91 | uuuuubk | uuuuuuu | 3.451 2.79e-04 |
| 92  Protein: TRA2B(Hs/Mm) | uuuuubk | uuuuuua | 3.382 3.60e-04 |
| Sequence Position | Motif | K-mer | Z-score P-value |
| 185 | aaguguu | agguggu | 2.105 1.76e-02 |
| 204 | aaguguu | agguauu | 1.916 2.77e-02 |
| 318 | aaguguu | gagcguu | 1.726 4.22e-02 |
| 427 | aaguguu | cuggguu | 2.105 1.76e-02 |
| 434 | aaguguu | aacuuug | 2.105 1.76e-02 |
| 443 | aaguguu | aaaugca | 1.916 2.77e-02 |
| 449  Protein: U2AF2(Hs/Mm) | aaguguu | aaguguu | 3.042 1.18e-03 |
| Sequence Position | Motif | K-mer | Z-score P-value |
| 91 | uuuuuyc | uuuuuuu | 3.352 4.01e-04 |
| 92 | uuuuuyc | uuuuuua | 3.314 4.60e-04 |
| 101 | uuuuuyc | cuuuucc | 3.200 6.87e-04 |
| 210 | uuuuuyc | ucuuuuc | 2.629 4.28e-03 |
| 211 | uuuuuyc | cuuuucc | 2.657 3.94e-03 |
| 256 | uuuuuyc | ucuuuuc | 2.048 2.03e-02 |
| 257 | uuuuuyc | cuuuucc | 2.076 1.89e-02 |
| Protein: ZC3H10(Hs/Mm) | |  |  |
| Sequence Position Motif | | K-mer | Z-score P-value |
| 169 ssagcgm  Protein: ZC3H14(Hs/Mm) | | ccagcgg | 2.397 8.26e-03 |
| Sequence Position Motif | | K-mer | Z-score P-value |
| 91 uuuduuu  Protein: ZCRB1(Hs/Mm) | | uuuuuuu | 2.856 2.15e-03 |
| Sequence Position Motif | | K-mer | Z-score P-value |
| 74 grhuuaa | | cacuuaa | 2.488 6.42e-03 |

************************************************************************************************

EXON3

==============================================================================

Protein: BRUNOL4(Hs/Mm)

Sequence Position Motif K-mer Z-score P-value

66 kgugukk uguuugu 3.703 1.07e-04

| Protein: BRUNOL5(Hs/Mm) | |  |  |
| --- | --- | --- | --- |
| Sequence Position | Motif | K-mer | Z-score P-value |
| 66 | ugugukk | uguuugu | 3.812 6.89e-05 |
| 70  Protein: CPEB2(Hs/Mm) | ugugukk | uguuugg | 3.613 1.51e-04 |
| Sequence Position | Motif | K-mer | Z-score P-value |
| 60 | chuuuuu | guuuuuu | 3.378 3.65e-04 |
| 76 | chuuuuu | guuuuuu | 3.378 3.65e-04 |
| 77 | chuuuuu | uuuuuuu | 3.449 2.81e-04 |
| 78 | chuuuuu | uuuuuuu | 3.449 2.81e-04 |
| 79 | chuuuuu | uuuuuuu | 3.449 2.81e-04 |
| 80 | chuuuuu | uuuuuuu | 3.449 2.81e-04 |
| 81 | chuuuuu | uuuuuuu | 3.449 2.81e-04 |
| 82 | chuuuuu | uuuuuuu | 3.449 2.81e-04 |
| 83 | chuuuuu | uuuuuuu | 3.449 2.81e-04 |
| 84 | chuuuuu | uuuuuuu | 3.449 2.81e-04 |
| 106  Protein: CPEB4(Hs/Mm) | chuuuuu | cuauuuu | 3.408 3.27e-04 |
| Sequence Position | Motif | K-mer | Z-score P-value |
| 54 | uuuuuu | auuuuu | 3.478 2.53e-04 |
| 60 | uuuuuu | guuuuu | 3.487 2.44e-04 |
| 61 | uuuuuu | uuuuuu | 3.522 2.14e-04 |
| 76 | uuuuuu | guuuuu | 3.487 2.44e-04 |
| 77 | uuuuuu | uuuuuu | 3.522 2.14e-04 |
| 78 | uuuuuu | uuuuuu | 3.522 2.14e-04 |
| 79 | uuuuuu | uuuuuu | 3.522 2.14e-04 |
| 80 | uuuuuu | uuuuuu | 3.522 2.14e-04 |
| 81 | uuuuuu | uuuuuu | 3.522 2.14e-04 |
| 82 | uuuuuu | uuuuuu | 3.522 2.14e-04 |
| 83 | uuuuuu | uuuuuu | 3.522 2.14e-04 |
| 84 | uuuuuu | uuuuuu | 3.522 2.14e-04 |
| 85 | uuuuuu | uuuuuu | 3.522 2.14e-04 |
| 92 | uuuuuu | auuuuu | 3.487 2.44e-04 |
| Protein: CUG-BP(Hs/Mm) | |  |  |
| Sequence Position Motif | | K-mer | Z-score P-value |
| 149 ugcug | | uccug | 1.823 3.42e-02 |
| 152 ugcug | | ugcuu | 2.044 2.05e-02 |
| 175 ugcug | | cgcug | 2.035 2.09e-02 |
| 198 ugcug | | ugccg | 2.142 1.61e-02 |
| 203 ugcug | | ggcug | 2.027 2.13e-02 |
| 210 ugcug  Protein: FUS(Hs/Mm) | | ugcuu | 2.000 2.27e-02 |
| Sequence Position Motif | | K-mer | Z-score P-value |
| 184 cgcgc | | ugcgc | 1.892 2.92e-02 |
| Protein: HNRNPA2B1(Hs/Mm) | |  |  |
| Sequence Position Motif | | K-mer | Z-score P-value |
| 160 duagggw  Protein: HNRNPC(Hs/Mm) | | caaggga | 2.333 9.82e-03 |
| Sequence Position Motif | | K-mer | Z-score P-value |
| 42 huuuuuk | | auguuuu | 3.205 6.75e-04 |
| 54 huuuuuk | | auuuuug | 3.714 1.02e-04 |
| 60 huuuuuk | | guuuuuu | 3.625 1.44e-04 |
| 61 huuuuuk | | uuuuuug | 3.518 2.17e-04 |
| 76 huuuuuk | | guuuuuu | 3.625 1.44e-04 |
| 77 huuuuuk | | uuuuuuu | 3.679 1.17e-04 |
| 78 huuuuuk | | uuuuuuu | 3.679 1.17e-04 |
| 79 huuuuuk | | uuuuuuu | 3.679 1.17e-04 |
| 80 huuuuuk | | uuuuuuu | 3.634 1.40e-04 |
| 81 huuuuuk | | uuuuuuu | 3.634 1.40e-04 |
| 82 huuuuuk | | uuuuuuu | 3.634 1.40e-04 |
| 83 huuuuuk | | uuuuuuu | 3.634 1.40e-04 |
| 84 huuuuuk | | uuuuuuu | 3.634 1.40e-04 |
| 85 huuuuuk | | uuuuuua | 3.339 4.20e-04 |
| 91 huuuuuk | | aauuuuu | 3.268 5.42e-04 |
| 92 huuuuuk  Protein: HNRNPCL1(Hs/Mm) | | auuuuua | 3.589 1.66e-04 |
| Sequence Position Motif | | K-mer | Z-score P-value |
| 42 huuuuuk | | auguuuu | 3.167 7.70e-04 |
| 54 huuuuuk | | auuuuug | 3.675 1.19e-04 |
| 60 huuuuuk | | guuuuuu | 3.605 1.56e-04 |
| 61 huuuuuk | | uuuuuug | 3.491 2.41e-04 |
| 76 huuuuuk | | guuuuuu | 3.605 1.56e-04 |
| 77 huuuuuk | | uuuuuuu | 3.684 1.15e-04 |
| 78 huuuuuk | | uuuuuuu | 3.684 1.15e-04 |
| 79 huuuuuk | | uuuuuuu | 3.684 1.15e-04 |
| 80 huuuuuk | | uuuuuuu | 3.667 1.23e-04 |
| 81 huuuuuk | | uuuuuuu | 3.667 1.23e-04 |
| 82 huuuuuk | | uuuuuuu | 3.667 1.23e-04 |
| 83 huuuuuk | | uuuuuuu | 3.667 1.23e-04 |
| 84 huuuuuk | | uuuuuuu | 3.667 1.23e-04 |
| 85 huuuuuk | | uuuuuua | 3.351 4.03e-04 |
| 91 huuuuuk | | aauuuuu | 3.272 5.34e-04 |
| 92 huuuuuk  Protein: HNRNPH2(Hs/Mm) | | auuuuua | 3.570 1.78e-04 |
| Sequence Position Motif | | K-mer | Z-score P-value |
| 141 gggaggg | | uggaggg | 2.567 5.13e-03 |
| 163 gggaggg | | gggaagg | 2.933 1.68e-03 |
| 164 gggaggg | | ggaaggg | 2.978 1.45e-03 |

Protein: HNRNPM(Hs/Mm)

| Sequence Position Motif | | K-mer | Z-score P-value |
| --- | --- | --- | --- |
| 49 gguugguu | | gggugauu | 4.127 1.84e-05 |
| 67 gguugguu | | guuuguuu | 4.222 1.21e-05 |
| 71 gguugguu  Protein: HNRNPU(Hs/Mm) | | guuugguu | 4.540 2.81e-06 |
| Sequence Position | Motif | K-mer | Z-score P-value |
| 43  Protein: HuR(Hs/Mm) | uguauug | uguuuug | 2.609 4.54e-03 |
| Sequence Position | Motif | K-mer | Z-score P-value |
| 42 | uukruuu | auguuuu | 3.495 2.37e-04 |
| 51 | uukruuu | gugauuu | 3.703 1.07e-04 |
| 57 | uukruuu | uuuguuu | 4.231 1.16e-05 |
| 58 | uukruuu | uuguuuu | 4.099 2.07e-05 |
| 64 | uukruuu | uuuguuu | 4.231 1.16e-05 |
| 68 | uukruuu | uuuguuu | 4.231 1.16e-05 |
| 73 | uukruuu | uugguuu | 4.352 6.75e-06 |
| 77 | uukruuu | uuuuuuu | 3.857 5.74e-05 |
| 78 | uukruuu | uuuuuuu | 3.857 5.74e-05 |
| 79 | uukruuu | uuuuuuu | 3.857 5.74e-05 |
| 80 | uukruuu | uuuuuuu | 3.857 5.74e-05 |
| 81 | uukruuu | uuuuuuu | 3.857 5.74e-05 |
| 82 | uukruuu | uuuuuuu | 3.857 5.74e-05 |
| 83 | uukruuu | uuuuuuu | 3.846 6.00e-05 |
| 84 | uukruuu | uuuuuuu | 3.835 6.28e-05 |
| 89  Protein: MBNL1(Hs/Mm) | uukruuu | uuaauuu | 3.868 5.49e-05 |
| Sequence Position | Motif | K-mer | Z-score P-value |
| 26 | ygcuky | cgcugg | 2.392 8.38e-03 |
| 36 | ygcuky | uucuuu | 2.480 6.57e-03 |
| 43 | ygcuky | uguuuu | 2.480 6.57e-03 |
| 52 | ygcuky | ugauuu | 2.451 7.12e-03 |
| 59 | ygcuky | uguuuu | 2.451 7.12e-03 |
| 74 | ygcuky | ugguuu | 2.294 1.09e-02 |
| 100 | ygcuky | cccuuu | 1.686 4.59e-02 |
| 137 | gcuugc | ggcuug | 2.013 2.21e-02 |
| 149 | ygcuky | uccugc | 2.480 6.57e-03 |
| 152 | ygcuky | ugcuuu | 3.108 9.42e-04 |
| 175 | ygcuky | cgcuga | 2.794 2.60e-03 |
| 186 | ygcuky | cgcucc | 2.804 2.52e-03 |
| 195 | ygcuky | cccugc | 2.804 2.52e-03 |
| 210  Protein: NOVA1(Hs/Mm) | ygcuky | ugcuuc | 3.108 9.42e-04 |
| Sequence Position | Motif | K-mer | Z-score P-value |
| 214 | ycay | ucau | 2.750 2.98e-03 |

| Protein: PCBP2(Hs/Mm) |  |  |  |
| --- | --- | --- | --- |
| Sequence Position | Motif | K-mer | Z-score P-value |
| 113 | ccyycch | cccuccc | 3.150 8.16e-04 |
| 114  Protein: PCBP3(Hs/Mm) | ccyycch | ccucccc | 3.188 7.16e-04 |
| Sequence Position | Motif | K-mer | Z-score P-value |
| 109  Protein: PTBP1(Hs/Mm) | uuuycc | uuuucc | 3.527 2.10e-04 |
| Sequence Position | Motif | K-mer | Z-score P-value |
| 36 | cucucu | uucuuu | 1.920 2.74e-02 |
| 37 | ucuu | ucuu | 2.723 3.23e-03 |
| 38 | cucucu | cuuuau | 1.911 2.80e-02 |
| 43 | ucuu | uguu | 2.161 1.53e-02 |
| 45 | ucuu | uuuu | 2.161 1.53e-02 |
| 55 | ucuu | uuuu | 2.161 1.53e-02 |
| 56 | ucuu | uuuu | 2.161 1.53e-02 |
| 59 | ucuu | uguu | 2.161 1.53e-02 |
| 61 | ucuu | uuuu | 2.161 1.53e-02 |
| 62 | ucuu | uuuu | 2.161 1.53e-02 |
| 100 | cucucu | cccuuu | 1.920 2.74e-02 |
| 102 | cucucu | cuuucu | 2.295 1.09e-02 |
| 104 | cucucu | uucuau | 1.920 2.74e-02 |
| 106 | cucucu | cuauuu | 1.929 2.69e-02 |
| 111 | cucucu | uucccu | 1.929 2.69e-02 |
| 113 | cucucu | cccucc | 1.929 2.69e-02 |
| 115 | cucucu | cucccc | 1.929 2.69e-02 |
| 131 | cucucu | ggcucu | 1.929 2.69e-02 |
| 133 | cucucu | cucugg | 1.929 2.69e-02 |
| 135 | cucucu | cuggcu | 1.929 2.69e-02 |
| 154 | cucucu | cuuucu | 2.259 1.19e-02 |
| 156 | cucucu | uucuca | 1.884 2.98e-02 |
| 186 | cucucu | cgcucc | 1.741 4.08e-02 |
| 191 | cucucu | cacucc | 1.741 4.08e-02 |
| 193  Protein: RALY(Hs/Mm) | cucucu | cucccu | 2.116 1.72e-02 |
| Sequence Position | Motif | K-mer | Z-score P-value |
| 43 | uuuuuub | uguuuug | 3.393 3.46e-04 |
| 54 | uuuuuub | auuuuug | 3.705 1.06e-04 |
| 55 | uuuuuub | uuuuugu | 3.491 2.41e-04 |
| 61 | uuuuuub | uuuuuug | 4.062 2.43e-05 |
| 62 | uuuuuub | uuuuugu | 3.500 2.33e-04 |
| 65 | uuuuuub | uuguuug | 3.554 1.90e-04 |
| 69 | uuuuuub | uuguuug | 3.554 1.90e-04 |
| 77 | uuuuuub | uuuuuuu | 3.955 3.83e-05 |
| 78 | uuuuuub | uuuuuuu | 3.955 3.83e-05 |
| 79 | uuuuuub | uuuuuuu | 3.955 3.83e-05 |

| 80 uuuuuub | | uuuuuuu | 3.955 3.83e-05 |
| --- | --- | --- | --- |
| 81 uuuuuub | | uuuuuuu | 3.955 3.83e-05 |
| 82 uuuuuub | | uuuuuuu | 3.955 3.83e-05 |
| 83 uuuuuub | | uuuuuuu | 3.955 3.83e-05 |
| 84 uuuuuub | | uuuuuuu | 3.955 3.83e-05 |
| 85 uuuuuub  Protein: RBM24(Hs/Mm) | | uuuuuua | 3.875 5.33e-05 |
| Sequence Position Motif | | K-mer | Z-score P-value |
| 48 wgwgugd  Protein: SAMD4A(Hs/Mm) | | uggguga | 2.286 1.11e-02 |
| Sequence Position | Motif | K-mer | Z-score P-value |
| 204  Protein: SRSF1(Hs/Mm) | gckgghm | gcuggau | 1.654 4.91e-02 |
| Sequence Position | Motif | K-mer | Z-score P-value |
| 142  Protein: SRSF2(Hs/Mm) | gragga | ggaggg | 2.280 1.13e-02 |
| Sequence Position | Motif | K-mer | Z-score P-value |
| 149 | ugcygyy | uccugcu | 2.495 6.30e-03 |
| 152 | ugcygyy | ugcuuuc | 2.495 6.30e-03 |
| 172  Protein: SRSF3(Hs/Mm) | ugcygyy | agccgcu | 2.717 3.29e-03 |
| Sequence Position | Motif | K-mer | Z-score P-value |
| 100 | cuckucy | cccuuuc | 2.000 2.27e-02 |
| 101 | cuckucy | ccuuucu | 2.000 2.27e-02 |
| 106 | cuckucy | cuauuuu | 2.000 2.27e-02 |
| 108 | cuckucy | auuuucc | 2.020 2.17e-02 |
| 113 | cuckucy | cccuccc | 2.020 2.17e-02 |
| 133 | cuckucy | cucuggc | 1.950 2.56e-02 |
| 148 | cuckucy | cuccugc | 1.680 4.65e-02 |
| 188 | cuckucy | cuccacu | 1.840 3.29e-02 |
| 191 | cuckucy | cacuccc | 1.840 3.29e-02 |
| 195  Protein: SRSF5(Hs/Mm) | cuckucy | cccugcc | 1.840 3.29e-02 |
| Sequence Position | Motif | K-mer | Z-score P-value |
| 132 | yywcwsg | gcucugg | 2.516 5.93e-03 |
| 155 | yywcwsg | uuucuca | 2.484 6.50e-03 |
| 157 | yywcwsg | ucucaag | 2.484 6.50e-03 |
| 190 | yywcwsg | ccacucc | 1.914 2.78e-02 |
| Protein: TARDBP(Hs/Mm) | |  |  |
| Sequence Position Motif | | K-mer | Z-score P-value |
| 39 ugugug | | uuuaug | 2.009 2.23e-02 |
| 41 ugugug | | uauguu | 2.202 1.38e-02 |

| 43 | ugugug | uguuuu | 2.202 1.38e-02 |
| --- | --- | --- | --- |
| 46 | ugugug | uuuggg | 2.303 1.06e-02 |
| 48 | ugugug | ugggug | 2.963 1.52e-03 |
| 55 | ugugug | uuuuug | 2.303 1.06e-02 |
| 57 | ugugug | uuuguu | 2.303 1.06e-02 |
| 59 | ugugug | uguuuu | 2.303 1.06e-02 |
| 62 | ugugug | uuuuug | 2.303 1.06e-02 |
| 64 | ugugug | uuuguu | 2.303 1.06e-02 |
| 66 | ugugug | uguuug | 2.963 1.52e-03 |
| 68 | ugugug | uuuguu | 2.303 1.06e-02 |
| 70  Protein: TIA1(Hs/Mm) | ugugug | uguuug | 2.963 1.52e-03 |
| Sequence Position | Motif | K-mer | Z-score P-value |
| 54 | uuuuubk | auuuuug | 3.716 1.01e-04 |
| 55 | uuuuubk | uuuuugu | 3.657 1.28e-04 |
| 61 | uuuuubk | uuuuuug | 4.029 2.80e-05 |
| 62 | uuuuubk | uuuuugu | 3.667 1.23e-04 |
| 77 | uuuuubk | uuuuuuu | 3.922 4.39e-05 |
| 78 | uuuuubk | uuuuuuu | 3.922 4.39e-05 |
| 79 | uuuuubk | uuuuuuu | 3.922 4.39e-05 |
| 80 | uuuuubk | uuuuuuu | 3.922 4.39e-05 |
| 81 | uuuuubk | uuuuuuu | 3.922 4.39e-05 |
| 82 | uuuuubk | uuuuuuu | 3.922 4.39e-05 |
| 83 | uuuuubk | uuuuuuu | 3.922 4.39e-05 |
| 84 | uuuuubk | uuuuuuu | 3.922 4.39e-05 |
| 85  Protein: TRA2B(Hs/Mm) | uuuuubk | uuuuuua | 3.794 7.41e-05 |
| Sequence Position | Motif | K-mer | Z-score P-value |
| 42 | aaguguu | auguuuu | 1.916 2.77e-02 |
| 67  Protein: U2AF2(Hs/Mm) | aaguguu | guuuguu | 1.716 4.31e-02 |
| Sequence Position | Motif | K-mer | Z-score P-value |
| 61 | uuuuuyc | uuuuuug | 3.495 2.37e-04 |
| 77 | uuuuuyc | uuuuuuu | 3.581 1.71e-04 |
| 78 | uuuuuyc | uuuuuuu | 3.581 1.71e-04 |
| 79 | uuuuuyc | uuuuuuu | 3.581 1.71e-04 |
| 80 | uuuuuyc | uuuuuuu | 3.581 1.71e-04 |
| 81 | uuuuuyc | uuuuuuu | 3.581 1.71e-04 |
| 82 | uuuuuyc | uuuuuuu | 3.581 1.71e-04 |
| 83 | uuuuuyc | uuuuuuu | 3.581 1.71e-04 |
| 84 | uuuuuyc | uuuuuuu | 3.581 1.71e-04 |
| 85 | uuuuuyc | uuuuuua | 3.495 2.37e-04 |
| 93 | uuuuuyc | uuuuuac | 3.505 2.28e-04 |
| 107 | uuuuuyc | uauuuuc | 3.267 5.43e-04 |
| 108 | uuuuuyc | auuuucc | 3.314 4.60e-04 |
| Protein: ZC3H14(Hs/Mm) | |  |  |
| Sequence Position | Motif | K-mer | Z-score P-value |
| 41 | uuuduuu | uauguuu | 3.443 2.88e-04 |
| 56 | uuuduuu | uuuuguu | 3.629 1.42e-04 |
| 57 | uuuduuu | uuuguuu | 4.237 1.13e-05 |
| 58 | uuuduuu | uuguuuu | 3.588 1.67e-04 |
| 63 | uuuduuu | uuuuguu | 3.629 1.42e-04 |
| 64 | uuuduuu | uuuguuu | 4.237 1.13e-05 |
| 68 | uuuduuu | uuuguuu | 4.237 1.13e-05 |
| 72 | uuuduuu | uuugguu | 3.835 6.28e-05 |
| 73 | uuuduuu | uugguuu | 3.794 7.41e-05 |
| 77 | uuuduuu | uuuuuuu | 4.062 2.43e-05 |
| 78 | uuuduuu | uuuuuuu | 4.062 2.43e-05 |
| 79 | uuuduuu | uuuuuuu | 4.062 2.43e-05 |
| 80 | uuuduuu | uuuuuuu | 4.062 2.43e-05 |
| 81 | uuuduuu | uuuuuuu | 4.062 2.43e-05 |
| 82 | uuuduuu | uuuuuuu | 4.062 2.43e-05 |
| 83 | uuuduuu | uuuuuuu | 4.041 2.66e-05 |
| 84 | uuuduuu | uuuuuuu | 4.041 2.66e-05 |
| 89 | uuuduuu | uuaauuu | 3.557 1.88e-04 |
| Protein: ZNF638(Hs/Mm) | |  |  |
| Sequence Position Motif | | K-mer | Z-score P-value |
| 34 bguusku | | aguucuu | 3.338 4.22e-04 |
| 66 bguusku | | uguuugu | 3.831 6.38e-05 |

************************************************************************************************

EXON4

==============================================================================

| Protein: CUG-BP(Hs/Mm) | |  |  |
| --- | --- | --- | --- |
| Sequence Position | Motif | K-mer | Z-score P-value |
| 52 | ugcug | cgcug | 2.044 2.05e-02 |
| 56 | ugcug | ggcug | 2.150 1.58e-02 |
| 77 | ugcug | cgcug | 2.212 1.35e-02 |
| 80 | ugcug | ugccg | 2.150 1.58e-02 |
| 102 | ugcug | cgcug | 2.186 1.44e-02 |
| 106 | ugcug | ggcug | 2.000 2.27e-02 |
| 110 | ugcug | ggcug | 1.965 2.47e-02 |
| 229 | ugcug | cgcug | 1.655 4.90e-02 |
| 232  Protein: FMR1(Hs/Mm) | ugcug | ugcuu | 1.655 4.90e-02 |
| Sequence Position | Motif | K-mer | Z-score P-value |
| 280  Protein: FUS(Hs/Mm) | kgacarg | ggaccag | 2.625 4.33e-03 |
| Sequence Position | Motif | K-mer | Z-score P-value |
| 50 cgcgc | | ggcgc | 2.494 6.32e-03 |
| 86 cgcgc | | cgcgc | 3.627 1.43e-04 |
| 94 cgcgc | | ggcgc | 3.651 1.31e-04 |
| 130 cgcgc | | ugcgc | 1.892 2.92e-02 |
| 252 cgcgc | | agcgc | 2.747 3.01e-03 |
| 273 cgcgc  Protein: FXR2(Hs/Mm) | | ggcgc | 1.771 3.83e-02 |
| Sequence Position Motif | | K-mer | Z-score P-value |
| 243 dgacrrr | | agacgag | 2.671 3.78e-03 |
| 280 dgacrrr  Protein: HNRNPA2B1(Hs/Mm) | | ggaccag | 3.000 1.35e-03 |
| Sequence Position Motif | | K-mer | Z-score P-value |
| 276 duagggw  Protein: HNRNPH2(Hs/Mm) | | gcaggga | 2.467 6.81e-03 |
| Sequence Position | Motif | K-mer | Z-score P-value |
| 168 | gggaggg | gggcggg | 3.078 1.04e-03 |
| 268  Protein: MBNL1(Hs/Mm) | gggaggg | ggcaggg | 2.522 5.83e-03 |
| Sequence Position | Motif | K-mer | Z-score P-value |
| 52 | ygcuky | cgcugg | 2.706 3.41e-03 |
| 74 | ygcuky | cgccgc | 2.863 2.10e-03 |
| 77 | ygcuky | cgcugc | 3.294 4.94e-04 |
| 80 | ygcuky | ugccgc | 2.863 2.10e-03 |
| 83 | ygcuky | cgccgc | 2.863 2.10e-03 |
| 102 | ygcuky | cgcugg | 2.863 2.10e-03 |
| 110 | ygcuky | ggcugu | 2.098 1.80e-02 |
| 194 | ygcuky | ggcuuu | 1.676 4.69e-02 |
| 229 | ygcuky | cgcugc | 3.304 4.77e-04 |
| 232 | ygcuky | ugcuuu | 3.304 4.77e-04 |
| 309  Protein: PPRC1(Hs/Mm) | ygcuky | cacugc | 2.039 2.07e-02 |
| Sequence Position | Motif | K-mer | Z-score P-value |
| 85  Protein: RBM4(Hs/Mm) | ssgcgcs | ccgcgcc | 3.590 1.65e-04 |
| Sequence Position | Motif | K-mer | Z-score P-value |
| 87 | gcgcgss | gcgccgg | 2.802 2.54e-03 |
| 133 | gcgcgss | gcccggg | 2.429 7.57e-03 |
| 168 | gcgcgss | gggcggg | 1.747 4.03e-02 |
| 170 | gcgcgss | gcggggg | 1.813 3.49e-02 |
| 255 | gcgcgss | gcacggg | 2.593 4.76e-03 |
| 274 | gcgcgss | gcgcagg | 3.044 1.17e-03 |

Protein: RBM8A(Hs/Mm)

| Sequence Position Motif | | K-mer | Z-score P-value |
| --- | --- | --- | --- |
| 131 rygcgcb  Protein: SAMD4A(Hs/Mm) | | gcgcccg | 3.027 1.23e-03 |
| Sequence Position | Motif | K-mer | Z-score P-value |
| 103 | gckgghm | gcuggcu | 2.704 3.43e-03 |
| 107  Protein: SRSF1(Hs/Mm) | gckgghm | gcuggcu | 2.704 3.43e-03 |
| Sequence Position | Motif | K-mer | Z-score P-value |
| 242  Protein: SRSF2(Hs/Mm) | crsmsgw | cagacga | 2.389 8.45e-03 |
| Sequence Position | Motif | K-mer | Z-score P-value |
| 49 | ugcygyy | uggcgcu | 2.737 3.10e-03 |
| 74 | ugcygyy | cgccgcu | 3.384 3.57e-04 |
| 77 | ugcygyy | cgcugcc | 3.293 4.96e-04 |
| 80 | ugcygyy | ugccgcc | 3.646 1.33e-04 |
| 110 | ugcygyy | ggcuguc | 2.343 9.56e-03 |
| 229  Protein: SRSF3(Hs/Mm) | ugcygyy | cgcugcu | 2.172 1.49e-02 |
| Sequence Position | Motif | K-mer | Z-score P-value |
| 216 | cuckucy | cucagcu | 1.850 3.22e-02 |
| 221 | cuckucy | cucgcca | 1.850 3.22e-02 |
| 229 | cuckucy | cgcugcu | 1.840 3.29e-02 |
| 294  Protein: SRSF5(Hs/Mm) | wcwwc | acaac | 2.676 3.73e-03 |
| Sequence Position | Motif | K-mer | Z-score P-value |
| 62 | yywcwsg | gcucugg | 1.882 2.99e-02 |
| 101 | yywcwsg | ccgcugg | 2.269 1.16e-02 |
| 115 | yywcwsg | ucgcacg | 2.204 1.38e-02 |
| 215 | yywcwsg | ccucagc | 1.925 2.71e-02 |
| 308  Protein: SRSF7(Hs/Mm) | yywcwsg | ccacugc | 1.828 3.38e-02 |
| Sequence Position | Motif | K-mer | Z-score P-value |
| 151 | wggacra | uggagaa | 1.957 2.52e-02 |
| Protein: TARDBP(Hs/Mm) | |  |  |
| Sequence Position Motif | | K-mer | Z-score P-value |
| 32 ugugug | | ugucug | 2.183 1.45e-02 |
| 45 ugugug | | ugccug | 2.156 1.55e-02 |
| 55 ugugug | | uggcug | 2.156 1.55e-02 |
| 64 ugugug | | ucuggg | 1.917 2.76e-02 |
| 66 ugugug | | ugggug | 2.294 1.09e-02 |

************************************************************************************************ EXON5

==============================================================================

| Protein: BRUNOL4(Hs/Mm) |  |  |
| --- | --- | --- |
| Sequence Position Motif | K-mer | Z-score P-value |
| 35 kgugukk  Protein: BRUNOL5(Hs/Mm) | ugugugu | 4.216 1.24e-05 |
| Sequence Position Motif | K-mer | Z-score P-value |
| 35 ugugukk  Protein: BRUNOL6(Hs/Mm) | ugugugu | 4.300 8.54e-06 |
| Sequence Position Motif | K-mer | Z-score P-value |
| 35 ugugdkg | ugugugu | 3.693 1.11e-04 |
| 58 ugugdkg  Protein: CPEB2(Hs/Mm) | uguggug | 4.013 3.00e-05 |
| Sequence Position Motif | K-mer | Z-score P-value |
| 45 chuuuuu  Protein: CPEB4(Hs/Mm) | cuuuuuc | 2.827 2.35e-03 |
| Sequence Position Motif | K-mer | Z-score P-value |
| 45 uuuuuu  Protein: CUG-BP(Hs/Mm) | cuuuuu | 2.730 3.17e-03 |
| Sequence Position Motif | K-mer | Z-score P-value |
| 60 ugcug | uggug | 2.035 2.09e-02 |
| 63 ugcug | ugcgg | 2.035 2.09e-02 |
| 73 ugcug  Protein: HNRNPC(Hs/Mm) | ugcgg | 2.035 2.09e-02 |
| Sequence Position Motif | K-mer | Z-score P-value |
| 45 huuuuuk  Protein: HNRNPCL1(Hs/Mm) | cuuuuuc | 2.366 8.99e-03 |
| Sequence Position Motif | K-mer | Z-score P-value |
| 45 huuuuuk  Protein: HNRNPH2(Hs/Mm) | cuuuuuc | 2.351 9.36e-03 |
| Sequence Position Motif | K-mer | Z-score P-value |
| 66 gggaggg  Protein: MBNL1(Hs/Mm) | gggaagg | 2.756 2.93e-03 |
| Sequence Position Motif | K-mer | Z-score P-value |
| 43 ygcuky | cgcuuu | 3.216 6.50e-04 |
| 55 ygcuky | cucugu | 2.706 3.41e-03 |
| 60 ygcuky | uggugc | 2.706 3.41e-03 |

| Protein: PTBP1(Hs/Mm) |  |  |  |
| --- | --- | --- | --- |
| Sequence Position | Motif | K-mer | Z-score P-value |
| 41 | cucucu | uucgcu | 2.205 1.37e-02 |
| 43 | cucucu | cgcuuu | 2.116 1.72e-02 |
| 45 | cucucu | cuuuuu | 1.929 2.69e-02 |
| 47 | cucucu | uuuucu | 1.920 2.74e-02 |
| 51 | cucucu | cuagcu | 1.920 2.74e-02 |
| 53 | cucucu | agcucu | 1.920 2.74e-02 |
| 55  Protein: RALY(Hs/Mm) | cucucu | cucugu | 2.295 1.09e-02 |
| Sequence Position | Motif | K-mer | Z-score P-value |
| 46  Protein: RBM24(Hs/Mm) | uuuuuub | uuuuucu | 2.384 8.56e-03 |
| Sequence Position | Motif | K-mer | Z-score P-value |
| 35  Protein: RBM38(Hs/Mm) | wgwgugd | ugugugu | 3.558 1.87e-04 |
| Sequence Position | Motif | K-mer | Z-score P-value |
| 34 | kkguguk | uugugug | 4.103 2.04e-05 |
| 36  Protein: RBM4(Hs/Mm) | kkguguk | guguguu | 4.090 2.16e-05 |
| Sequence Position | Motif | K-mer | Z-score P-value |
| 62 | gcgcgss | gugcggg | 1.681 4.64e-02 |
| 72  Protein: RBM5(Hs/Mm) | gcgcgss | gugcggg | 1.681 4.64e-02 |
| Sequence Position | Motif | K-mer | Z-score P-value |
| 68  Protein: SRSF3(Hs/Mm) | garggwr | gaaggug | 2.269 1.16e-02 |
| Sequence Position | Motif | K-mer | Z-score P-value |
| 43 | cuckucy | cgcuuuu | 1.860 3.14e-02 |
| 45 | cuckucy | cuuuuuc | 1.860 3.14e-02 |
| 46  Protein: SRSF5(Hs/Mm) | cuckucy | uuuuucu | 1.860 3.14e-02 |
| Sequence Position | Motif | K-mer | Z-score P-value |
| 48 | yywcwsg | uuucuag | 2.204 1.38e-02 |
| 56 | yywcwsg | ucugugg | 2.086 1.85e-02 |
| Protein: TARDBP(Hs/Mm) | |  |  |
| Sequence Position Motif | | K-mer | Z-score P-value |
| 33 ugugug | | auugug | 2.743 3.04e-03 |
| 35 ugugug | | ugugug | 3.872 5.40e-05 |
| 37 ugugug | | uguguu | 3.486 2.45e-04 |
| 39 ugugug | | uguucg | 2.752 2.96e-03 |
| 56 | ugugug | ucugug | 3.349 4.06e-04 |
| 58 | ugugug | uguggu | 2.679 3.69e-03 |
| 61 | ugugug | ggugcg | 2.193 1.42e-02 |
| 63 | ugugug | ugcggg | 2.000 2.27e-02 |
| 71 | ugugug | ggugcg | 2.000 2.27e-02 |
| 73  Protein: TIA1(Hs/Mm) | ugugug | ugcggg | 2.009 2.23e-02 |
| Sequence Position | Motif | K-mer | Z-score P-value |
| 46  Protein: TRA2B(Hs/Mm) | uuuuubk | uuuuucu | 2.490 6.39e-03 |
| Sequence Position | Motif | K-mer | Z-score P-value |
| 36  Protein: U2AF2(Hs/Mm) | aaguguu | guguguu | 1.821 3.43e-02 |
| Sequence Position | Motif | K-mer | Z-score P-value |
| 45 | uuuuuyc | cuuuuuc | 2.400 8.20e-03 |
| 46 | uuuuuyc | uuuuucu | 2.286 1.11e-02 |
| Protein: ZNF638(Hs/Mm) | |  |  |
| Sequence Position Motif | | K-mer | Z-score P-value |
| 39 bguusku | | uguucgc | 2.415 7.87e-03 |

************************************************************************************************

EXON6

==============================================================================

| Protein: BRUNOL4(Hs/Mm) |  |  |
| --- | --- | --- |
| Sequence Position Motif | K-mer | Z-score P-value |
| 332 kgugukk | uuugugu | 1.851 3.21e-02 |
| 334 kgugukk  Protein: BRUNOL5(Hs/Mm) | ugugugg | 1.986 2.35e-02 |
| Sequence Position Motif | K-mer | Z-score P-value |
| 332 ugugukk | uuugugu | 1.875 3.04e-02 |
| 334 ugugukk  Protein: BRUNOL6(Hs/Mm) | ugugugg | 2.025 2.14e-02 |
| Sequence Position Motif | K-mer | Z-score P-value |
| 334 ugugdkg  Protein: CUG-BP(Hs/Mm) | ugugugg | 1.773 3.81e-02 |
| Sequence Position Motif | K-mer | Z-score P-value |
| 52 ugcug | cgcug | 2.044 2.05e-02 |
| 56 ugcug | ggcug | 2.150 1.58e-02 |
| 77 ugcug | cgcug | 2.212 1.35e-02 |
| 80 ugcug | ugccg | 2.150 1.58e-02 |

| 102 | ugcug | cgcug | 2.186 1.44e-02 |
| --- | --- | --- | --- |
| 106 | ugcug | ggcug | 2.000 2.27e-02 |
| 110 | ugcug | ggcug | 1.965 2.47e-02 |
| 231 | ugcug | cgcug | 1.655 4.90e-02 |
| 234  Protein: FMR1(Hs/Mm) | ugcug | ugcuu | 1.655 4.90e-02 |
| Sequence Position | Motif | K-mer | Z-score P-value |
| 282  Protein: FUS(Hs/Mm) | kgacarg | ggaccag | 2.625 4.33e-03 |
| Sequence Position | Motif | K-mer | Z-score P-value |
| 50 | cgcgc | ggcgc | 2.494 6.32e-03 |
| 86 | cgcgc | cgcgc | 3.627 1.43e-04 |
| 94 | cgcgc | ggcgc | 3.651 1.31e-04 |
| 130 | cgcgc | ugcgc | 1.892 2.92e-02 |
| 174 | cgcgc | ggcgc | 3.289 5.03e-04 |
| 176 | cgcgc | cgcgc | 3.289 5.03e-04 |
| 254 | cgcgc | agcgc | 2.747 3.01e-03 |
| 275  Protein: FXR2(Hs/Mm) | cgcgc | ggcgc | 1.771 3.83e-02 |
| Sequence Position | Motif | K-mer | Z-score P-value |
| 245 | dgacrrr | agacgag | 2.671 3.78e-03 |
| 282 | dgacrrr | ggaccag | 3.000 1.35e-03 |
| Protein: HNRNPA2B1(Hs/Mm) | |  |  |
| Sequence Position Motif | | K-mer | Z-score P-value |
| 278 duagggw  Protein: HNRNPH2(Hs/Mm) | | gcaggga | 2.467 6.81e-03 |
| Sequence Position Motif | | K-mer | Z-score P-value |
| 270 gggaggg  Protein: MBNL1(Hs/Mm) | | ggcaggg | 2.522 5.83e-03 |
| Sequence Position Motif | | K-mer | Z-score P-value |
| 52 ygcuky | | cgcugg | 2.706 3.41e-03 |
| 74 ygcuky | | cgccgc | 2.863 2.10e-03 |
| 77 ygcuky | | cgcugc | 3.294 4.94e-04 |
| 80 ygcuky | | ugccgc | 2.863 2.10e-03 |
| 83 ygcuky | | cgccgc | 2.863 2.10e-03 |
| 102 ygcuky | | cgcugg | 2.863 2.10e-03 |
| 110 ygcuky | | ggcugu | 2.098 1.80e-02 |
| 147 gcuugc | | ggcuug | 1.857 3.17e-02 |
| 196 ygcuky | | ggcuuu | 1.686 4.59e-02 |
| 231 ygcuky | | cgcugc | 3.304 4.77e-04 |
| 234 ygcuky | | ugcuuu | 3.304 4.77e-04 |
| 311 ygcuky | | cacugc | 2.039 2.07e-02 |
| 329 ygcuky | | agcuuu | 2.069 1.93e-02 |

| Protein: PPRC1(Hs/Mm) |  |  |  |
| --- | --- | --- | --- |
| Sequence Position | Motif | K-mer | Z-score P-value |
| 85 | ssgcgcs | ccgcgcc | 3.590 1.65e-04 |
| 173 | ssgcgcs | cggcgcg | 2.000 2.27e-02 |
| 175  Protein: RBM24(Hs/Mm) | ssgcgcs | gcgcgcc | 2.077 1.89e-02 |
| Sequence Position | Motif | K-mer | Z-score P-value |
| 334  Protein: RBM38(Hs/Mm) | wgwgugd | ugugugg | 2.584 4.88e-03 |
| Sequence Position | Motif | K-mer | Z-score P-value |
| 333  Protein: RBM4(Hs/Mm) | kkguguk | uugugug | 2.821 2.39e-03 |
| Sequence Position | Motif | K-mer | Z-score P-value |
| 87 | gcgcgss | gcgccgg | 2.802 2.54e-03 |
| 133 | gcgcgss | gcccggg | 2.429 7.57e-03 |
| 175 | gcgcgss | gcgcgcc | 1.681 4.64e-02 |
| 257 | gcgcgss | gcacggg | 2.593 4.76e-03 |
| 276  Protein: RBM8A(Hs/Mm) | gcgcgss | gcgcagg | 3.044 1.17e-03 |
| Sequence Position | Motif | K-mer | Z-score P-value |
| 131 | rygcgcb | gcgcccg | 3.027 1.23e-03 |
| 175 | rygcgcb | gcgcgcc | 1.986 2.35e-02 |
| Protein: SAMD4A(Hs/Mm) | |  |  |
| Sequence Position Motif | | K-mer | Z-score P-value |
| 103 gckgghm | | gcuggcu | 2.704 3.43e-03 |
| 107 gckgghm  Protein: SRSF1(Hs/Mm) | | gcuggcu | 2.704 3.43e-03 |
| Sequence Position Motif | | K-mer | Z-score P-value |
| 244 crsmsgw  Protein: SRSF2(Hs/Mm) | | cagacga | 2.389 8.45e-03 |
| Sequence Position Motif | | K-mer | Z-score P-value |
| 49 ugcygyy | | uggcgcu | 2.737 3.10e-03 |
| 74 ugcygyy | | cgccgcu | 3.384 3.57e-04 |
| 77 ugcygyy | | cgcugcc | 3.293 4.96e-04 |
| 80 ugcygyy | | ugccgcc | 3.646 1.33e-04 |
| 110 ugcygyy | | ggcuguc | 2.343 9.56e-03 |
| 231 ugcygyy  Protein: SRSF3(Hs/Mm) | | cgcugcu | 2.172 1.49e-02 |
| Sequence Position Motif | | K-mer | Z-score P-value |
| 181 cuckucy | | caccucc | 1.660 4.85e-02 |
| 184 | cuckucy | cuccgcc | 1.660 4.85e-02 |
| 218 | cuckucy | cucagcu | 1.850 3.22e-02 |
| 223 | cuckucy | cucgcca | 1.850 3.22e-02 |
| 231 | cuckucy | cgcugcu | 1.840 3.29e-02 |
| 296  Protein: SRSF5(Hs/Mm) | wcwwc | acaac | 2.676 3.73e-03 |
| Sequence Position | Motif | K-mer | Z-score P-value |
| 62 | yywcwsg | gcucugg | 1.882 2.99e-02 |
| 101 | yywcwsg | ccgcugg | 2.269 1.16e-02 |
| 115 | yywcwsg | ucgcacg | 2.204 1.38e-02 |
| 217 | yywcwsg | ccucagc | 1.925 2.71e-02 |
| 310  Protein: SRSF7(Hs/Mm) | yywcwsg | ccacugc | 1.828 3.38e-02 |
| Sequence Position | Motif | K-mer | Z-score P-value |
| 151 | wggacra | uggagaa | 1.957 2.52e-02 |
| Protein: TARDBP(Hs/Mm) | |  |  |
| Sequence Position Motif | | K-mer | Z-score P-value |
| 32 ugugug | | ugucug | 2.183 1.45e-02 |
| 45 ugugug | | ugccug | 2.156 1.55e-02 |
| 55 ugugug | | uggcug | 2.156 1.55e-02 |
| 64 ugugug | | ucuggg | 1.917 2.76e-02 |
| 66 ugugug | | ugggug | 2.294 1.09e-02 |
| 332 ugugug | | uuugug | 3.110 9.35e-04 |
| 334 ugugug | | ugugug | 3.495 2.37e-04 |
| 336 ugugug | | ugugga | 2.541 5.53e-03 |

************************************************************************************************

EXON7

==============================================================================

| Protein: MATR3(Hs/Mm) |  |  |  |
| --- | --- | --- | --- |
| Sequence Position | Motif | K-mer | Z-score P-value |
| 79  Protein: MBNL1(Hs/Mm) | maucuur | auucuug | 1.649 4.96e-02 |
| Sequence Position | Motif | K-mer | Z-score P-value |
| 34 | ygcuky | agcuuc | 1.686 4.59e-02 |
| 144 | ygcuky | cucugu | 2.088 1.84e-02 |
| 163  Protein: PTBP1(Hs/Mm) | ygcuky | cucuuu | 2.088 1.84e-02 |
| Sequence Position | Motif | K-mer | Z-score P-value |
| 35 | ucuu | gcuu | 2.098 1.80e-02 |
| 38 | ucuu | ucuu | 2.580 4.94e-03 |
| 69 | ucuu | acuu | 1.884 2.98e-02 |

| 77 | ucuu | ucau | 1.884 2.98e-02 |
| --- | --- | --- | --- |
| 81 | ucuu | ucuu | 2.580 4.94e-03 |
| 105 | ucuu | ucug | 1.884 2.98e-02 |
| 140 | cucucu | cccucu | 3.304 4.77e-04 |
| 142 | cucucu | cucucu | 3.679 1.17e-04 |
| 143 | ucuu | ucuc | 2.027 2.13e-02 |
| 144 | cucucu | cucugu | 3.304 4.77e-04 |
| 145 | ucuu | ucug | 2.027 2.13e-02 |
| 146 | cucucu | cugucc | 2.607 4.57e-03 |
| 161 | cucucu | accucu | 2.607 4.57e-03 |
| 163 | cucucu | cucuuu | 3.304 4.77e-04 |
| 164 | ucuu | ucuu | 2.580 4.94e-03 |
| 174  Protein: RBM42(Hs/Mm) | ucuu | uccu | 2.027 2.13e-02 |
| Sequence Position | Motif | K-mer | Z-score P-value |
| 61  Protein: RBM6(Hs/Mm) | aacuamg | aacuagg | 2.122 1.69e-02 |
| Sequence Position | Motif | K-mer | Z-score P-value |
| 151  Protein: SRSF1(Hs/Mm) | hauccar | cauccag | 4.183 1.44e-05 |
| Sequence Position | Motif | K-mer | Z-score P-value |
| 179  Protein: SRSF3(Hs/Mm) | crsmsgw | cagagga | 2.389 8.45e-03 |
| Sequence Position | Motif | K-mer | Z-score P-value |
| 34 | cuckucy | agcuucu | 1.820 3.44e-02 |
| 37 | cuckucy | uucuuac | 1.690 4.55e-02 |
| 54 | wcwwc | ucaac | 2.902 1.85e-03 |
| 117 | cuckucy | caggucc | 1.680 4.65e-02 |
| 129 | cuckucy | cuuguau | 1.860 3.14e-02 |
| 144 | cuckucy | cucuguc | 1.950 2.56e-02 |
| 160 | cuckucy | caccucu | 1.950 2.56e-02 |
| 163 | cuckucy | cucuuua | 1.950 2.56e-02 |
| 173 | cuckucy | auccucc | 1.840 3.29e-02 |
| 203  Protein: SRSF5(Hs/Mm) | wcwwc | ucauc | 2.431 7.53e-03 |
| Sequence Position | Motif | K-mer | Z-score P-value |
| 40 | yywcwsg | uuacaag | 1.914 2.78e-02 |
| 86 | yywcwsg | ucacugg | 2.656 3.95e-03 |
| 114 | yywcwsg | cuccagg | 2.086 1.85e-02 |
| 123 | yywcwsg | ccacagc | 2.559 5.25e-03 |
| 141 | yywcwsg | ccucucu | 2.581 4.93e-03 |
| 143 | yywcwsg | ucucugu | 2.581 4.93e-03 |
| 165 | yywcwsg | cuuuagg | 2.602 4.63e-03 |
| 178 | yywcwsg | ccagagg | 2.312 1.04e-02 |
| Protein: TRA2A(Hs/Mm) |  |  |  |
| Sequence Position | Motif | K-mer | Z-score P-value |
| 178 | gaagaggaag | ccagaggaag | 2.011 2.22e-02 |
| 184  Protein: TRA2B(Hs/Mm) | gaagaggaag | gaagugaaag | 2.011 2.22e-02 |
| Sequence Position | Motif | K-mer | Z-score P-value |
| 185 | aaguguu | aagugaa | 2.568 5.11e-03 |
| 189 | aaguguu | gaaaguu | 1.916 2.77e-02 |
| 190 | aaguguu | aaaguuu | 1.916 2.77e-02 |
| 191 | aaguguu | aaguuuu | 2.853 2.17e-03 |
| 209 | aaguguu | gggaguu | 2.147 1.59e-02 |

************************************************************************************************

EXON8

==============================================================================

| Protein: BRUNOL4(Hs/Mm) |  |  |
| --- | --- | --- |
| Sequence Position Motif | K-mer | Z-score P-value |
| 58 kgugukk | ugugugg | 1.676 4.69e-02 |
| 216 kgugukk | ggugugu | 4.027 2.82e-05 |
| 218 kgugukk | ugugugu | 4.135 1.77e-05 |
| 220 kgugukk  Protein: BRUNOL5(Hs/Mm) | ugugucu | 3.784 7.72e-05 |
| Sequence Position Motif | K-mer | Z-score P-value |
| 58 ugugukk | ugugugg | 2.575 5.01e-03 |
| 216 ugugukk | ggugugu | 4.062 2.43e-05 |
| 218 ugugukk | ugugugu | 4.250 1.07e-05 |
| 220 ugugukk  Protein: BRUNOL6(Hs/Mm) | ugugucu | 3.912 4.58e-05 |
| Sequence Position Motif | K-mer | Z-score P-value |
| 58 ugugdkg | ugugugg | 2.800 2.56e-03 |
| 213 ugugdkg | uguggug | 3.533 2.05e-04 |
| 218 ugugdkg  Protein: CUG-BP(Hs/Mm) | ugugugu | 3.227 6.25e-04 |
| Sequence Position Motif | K-mer | Z-score P-value |
| 210 ugcug | ggcug | 1.779 3.76e-02 |
| 215 ugcug | uggug | 1.779 3.76e-02 |
| 241 ugcug | agcug | 2.407 8.04e-03 |
| 256 ugcug | ugcug | 2.593 4.76e-03 |
| 265 ugcug  Protein: HNRNPC(Hs/Mm) | uggug | 2.150 1.58e-02 |
| Sequence Position Motif | K-mer | Z-score P-value |

| 152 huuuuuk  Protein: HNRNPCL1(Hs/Mm) | auguuuu | 1.777 3.78e-02 |
| --- | --- | --- |
| Sequence Position Motif | K-mer | Z-score P-value |
| 152 huuuuuk  Protein: HNRNPF(Hs/Mm) | auguuuu | 1.763 3.89e-02 |
| Sequence Position Motif | K-mer | Z-score P-value |
| 210 gggug | ggcug | 2.062 1.96e-02 |
| 215 gggug | uggug | 2.062 1.96e-02 |
| 217 gggug | gugug | 2.062 1.96e-02 |
| 219 gggug | gugug | 2.062 1.96e-02 |
| 237 gugkau  Protein: HNRNPL(Hs/Mm) | guggag | 2.893 1.91e-03 |
| Sequence Position Motif | K-mer | Z-score P-value |
| 168 amayama | ccacaca | 3.493 2.39e-04 |
| 170 amayama  Protein: HNRPLL(Hs/Mm) | acacacu | 3.440 2.91e-04 |
| Sequence Position Motif | K-mer | Z-score P-value |
| 146 rcahaca | ccauaca | 3.557 1.88e-04 |
| 166 rcahaca | acccaca | 3.519 2.17e-04 |
| 168 rcahaca | ccacaca | 3.696 1.10e-04 |
| 170 rcahaca  Protein: HuR(Hs/Mm) | acacacu | 3.519 2.17e-04 |
| Sequence Position Motif | K-mer | Z-score P-value |
| 152 uukruuu | auguuuu | 2.099 1.79e-02 |
| 157 uukruuu  Protein: KHDRBS3(Hs/Mm) | uugguau | 2.505 6.12e-03 |
| Sequence Position Motif | K-mer | Z-score P-value |
| 271 auaaav  Protein: MBNL1(Hs/Mm) | cauaaa | 1.689 4.56e-02 |
| Sequence Position Motif | K-mer | Z-score P-value |
| 153 ygcuky | uguuuu | 2.039 2.07e-02 |
| 173 ygcuky | cacuuu | 2.284 1.12e-02 |
| 194 ygcuky | ugcauc | 2.392 8.38e-03 |
| 210 ygcuky | ggcugu | 2.451 7.12e-03 |
| 215 ygcuky | uggugu | 2.451 7.12e-03 |
| 225 ygcuky | cucugc | 2.402 8.15e-03 |
| 230 ygcuky | cgccuc | 2.402 8.15e-03 |
| 256 ygcuky | ugcuga | 1.990 2.33e-02 |
| 265 ygcuky  Protein: NOVA1(Hs/Mm) | uggugu | 1.686 4.59e-02 |
| Sequence Position Motif | K-mer | Z-score P-value |

| 168 ycay  Protein: PTBP1(Hs/Mm) | | ccac | 2.750 2.98e-03 |
| --- | --- | --- | --- |
| Sequence Position Motif | | K-mer | Z-score P-value |
| 221 cucucu | | gugucu | 1.830 3.36e-02 |
| 223 cucucu | | gucucu | 2.205 1.37e-02 |
| 225 cucucu | | cucugc | 1.830 3.36e-02 |
| 233 cucucu  Protein: RBFOX1(Hs/Mm) | | cucagu | 1.830 3.36e-02 |
| Sequence Position | Motif | K-mer | Z-score P-value |
| 26 | wgcaugm | uggaugc | 1.763 3.89e-02 |
| 30 | wgcaugm | ugcaagc | 2.855 2.15e-03 |
| 139 | wgcaugm | ugcaugu | 2.145 1.60e-02 |
| 194  Protein: RBM24(Hs/Mm) | wgcaugm | ugcaucc | 2.592 4.77e-03 |
| Sequence Position | Motif | K-mer | Z-score P-value |
| 216 | wgwgugd | ggugugu | 2.857 2.14e-03 |
| 218  Protein: RBM38(Hs/Mm) | wgwgugd | ugugugu | 2.987 1.41e-03 |
| Sequence Position | Motif | K-mer | Z-score P-value |
| 57 | kkguguk | augugug | 3.115 9.20e-04 |
| 215 | kkguguk | uggugug | 3.974 3.53e-05 |
| 217 | kkguguk | gugugug | 4.333 7.35e-06 |
| 219  Protein: RBM41(Hs/Mm) | kkguguk | guguguc | 4.051 2.55e-05 |
| Sequence Position | Motif | K-mer | Z-score P-value |
| 148  Protein: SFPQ(Hs/Mm) | wuacwuk | auacaug | 2.617 4.44e-03 |
| Sequence Position | Motif | K-mer | Z-score P-value |
| 214  Protein: SRSF1(Hs/Mm) | kurrukk | guggugu | 1.704 4.42e-02 |
| Sequence Position | Motif | K-mer | Z-score P-value |
| 238  Protein: SRSF3(Hs/Mm) | ugrwgvh | uggagcu | 2.733 3.14e-03 |
| Sequence Position | Motif | K-mer | Z-score P-value |
| 73 | cuckucy | guuuucu | 1.680 4.65e-02 |
| 93 | cuckucy | cuaaucc | 1.860 3.14e-02 |
| 99 | cuckucy | cucguua | 1.680 4.65e-02 |
| 125 | wcwwc | ucuac | 2.431 7.53e-03 |
| 162 | cuckucy | aucuacc | 1.850 3.22e-02 |
| 163 | wcwwc | ucuac | 2.431 7.53e-03 |
| 173 | cuckucy | cacuuuc | 1.680 4.65e-02 |

| Protein: SRSF5(Hs/Mm) | |  |  |
| --- | --- | --- | --- |
| Sequence Position Motif | | K-mer | Z-score P-value |
| 50 yywcwsg | | caacugg | 2.086 1.85e-02 |
| 75 yywcwsg | | uuucucc | 2.462 6.91e-03 |
| 86 yywcwsg | | gcucagg | 2.269 1.16e-02 |
| 168 yywcwsg | | ccacaca | 2.312 1.04e-02 |
| 176 yywcwsg | | uuucaug | 2.484 6.50e-03 |
| 198 yywcwsg | | ucccagg | 2.204 1.38e-02 |
| 224 yywcwsg | | ucucugc | 2.398 8.24e-03 |
| 232 yywcwsg | | ccucagu | 2.462 6.91e-03 |
| 234 yywcwsg  Protein: SRSF7(Hs/Mm) | | ucagugg | 2.462 6.91e-03 |
| Sequence Position Motif | | K-mer | Z-score P-value |
| 46 wggacra  Protein: SRSF9(Hs/Mm) | | aggacaa | 3.436 2.95e-04 |
| Sequence Position Motif | | K-mer | Z-score P-value |
| 63 kgrwgsm  Protein: TARDBP(Hs/Mm) | | ggaagca | 2.451 7.12e-03 |
| Sequence Position | Motif | K-mer | Z-score P-value |
| 54 | ugugug | uggaug | 2.294 1.09e-02 |
| 56 | ugugug | gaugug | 2.339 9.67e-03 |
| 58 | ugugug | ugugug | 3.128 8.80e-04 |
| 60 | ugugug | ugugga | 2.367 8.97e-03 |
| 81 | ugugug | caugug | 2.339 9.67e-03 |
| 83 | ugugug | ugugcu | 2.284 1.12e-02 |
| 192 | ugugug | ugugca | 2.009 2.23e-02 |
| 211 | ugugug | gcugug | 2.679 3.69e-03 |
| 213 | ugugug | uguggu | 2.679 3.69e-03 |
| 216 | ugugug | ggugug | 3.349 4.06e-04 |
| 218 | ugugug | ugugug | 3.725 9.77e-05 |
| 220 | ugugug | uguguc | 3.349 4.06e-04 |
| 222 | ugugug | ugucuc | 2.679 3.69e-03 |
| 224 | ugugug | ucucug | 2.679 3.69e-03 |
| 226 | ugugug | ucugcg | 2.679 3.69e-03 |
| 234  Protein: TRA2B(Hs/Mm) | ugugug | ucagug | 2.679 3.69e-03 |
| Sequence Position | Motif | K-mer | Z-score P-value |
| 33 | aaguguu | aagcgua | 2.526 5.77e-03 |
| 57 | aaguguu | augugug | 2.474 6.68e-03 |
| 71 | aaguguu | caguuuu | 2.295 1.09e-02 |
| 82 | aaguguu | augugcu | 2.105 1.76e-02 |
| 132 | aaguguu | aaugggu | 1.768 3.85e-02 |
| 133 | aaguguu | auggguu | 2.432 7.51e-03 |
| 150 | aaguguu | acauguu | 2.105 1.76e-02 |
| 152 | aaguguu | auguuuu | 2.105 1.76e-02 |
| 262 | aaguguu | aaauggu | 1.905 2.84e-02 |
| 273  Protein: TUT1(Hs/Mm) | aaguguu | uaaaguu | 1.716 4.31e-02 |
| Sequence Position | Motif | K-mer | Z-score P-value |
| 104  Protein: U2AF2(Hs/Mm) | mrauacu | uaauacu | 2.590 4.80e-03 |
| Sequence Position | Motif | K-mer | Z-score P-value |
| 74 | uuuuuyc | uuuucuc | 2.448 7.18e-03 |

************************************************************************************************
